# Frailty does not cause all frail symptoms: United States Health and Retirement Study

**DOI:** 10.1371/journal.pone.0272289

**Published:** 2022-11-02

**Authors:** Yi-Sheng Chao, Chao-Jung Wu, June Y. T. Po, Shih-Yu Huang, Hsing-Chien Wu, Hui-Ting Hsu, Yen-Po Cheng, Yi-Chun Lai, Wei-Chih Chen

**Affiliations:** 1 Independent Researcher, Montreal, Canada; 2 Université du Québec à Montréal, Montreal, Canada; 3 Natural Resources Institute, University of Greenwich, London, United Kingdom; 4 Department of Anesthesiology, Shuang Ho Hospital, Taipei Medical University, New Taipei City, Taiwan; 5 Department of Anesthesiology, School of Medicine, College of Medicine, Taipei Medical University, Taipei, Taiwan; 6 Taipei Hospital, Ministry of Health and Welfare, New Taipei City, Taiwan; 7 Changhua Christian Hospital, Changhua, Taiwan; 8 National Yang-Ming University Hospital, Yilan, Taiwan; 9 Department of Chest Medicine, Taipei Veterans General Hospital, Institute of Emergency and Critical Care Medicine, National Yang-Ming University, Taipei City, Taiwan; Public Library of Science, UNITED KINGDOM

## Abstract

**Background:**

Frailty is associated with major health outcomes. However, the relationships between frailty and frailty symptoms haven’t been well studied. This study aims to show the associations between frailty and frailty symptoms.

**Methods:**

The Health and Retirement Study (HRS) is an ongoing longitudinal biannual survey in the United States. Three of the most used frailty diagnoses, defined by the Functional Domains Model, the Burden Model, and the Biologic Syndrome Model, were reproduced according to previous studies. The associations between frailty statuses and input symptoms were assessed using odds ratios and correlation coefficients.

**Results:**

The sample sizes, mean ages, and frailty prevalence matched those reported in previous studies. Frailty statuses were weakly correlated with each other (coefficients = 0.19 to 0.38, p < 0.001 for all). There were 49 input symptoms identified by these three models. Frailty statuses defined by the three models were not significantly correlated with one or two symptoms defined by the same models (p > 0.05 for all). One to six symptoms defined by the other two models were not significantly correlated with each of the three frailty statuses (p > 0.05 for all). Frailty statuses were significantly correlated with their own bias variables (p < 0.05 for all).

**Conclusion:**

Frailty diagnoses lack significant correlations with some of their own frailty symptoms and some of the frailty symptoms defined by the other two models. This finding raises questions like whether the frailty symptoms lacking significant correlations with frailty statuses could be included to diagnose frailty and whether frailty exists and causes frailty symptoms.

## Introduction

Frailty is a syndrome and can be diagnosed with composite criteria that consist of various frailty symptoms [1–3]. Frailty is often characterized by age-related symptoms, such as declines in physical and cognitive functioning. It has been considered significant for the prediction of major health outcomes, such as falls, surgical outcomes, and mortality [2, 3]. By aggregating information from multiple symptoms, frailty index scores can be assigned to individuals [2, 3]. Frailty status can then be derived by applying theoretical thresholds to frailty index scores [2, 3]. Three of the most commonly used frailty indices require 4 to 70 input domains or symptoms for the diagnosis of frailty [2].

Ideally, pathological changes or underlying health conditions are expected to cause or lead to significant increases in symptom occurrence. The increased frequency of symptom development can be used to make diagnoses that serve as proxy measures to the pathological changes{please add new reference: DOI:10.1038/s41598-022-14826-2}. For example, frailty has been recognized as a cause of disability, independent of clinical conditions [4]. Other researchers also found that frailty can lead to symptoms, particularly mental symptoms [5, 6] and fatigue [7]. Frailty has been confirmed using multiple frailty symptoms and considered a diagnosis that represent low physical reserve [8]. However, the effects of frailty on the development of frailty symptoms (those used to diagnose frailty) have not been well discussed. Instead, frailty has been described and defined differently [1]. Some studies have shown that how frailty is diagnosed seems far from ideal and lacks pathological confirmation [2]. Additionally, researchers have confirmed notable differences in the frail patients identified between the three frailty models [1].

In other words, whether frailty causes frailty symptoms remains unclear and whether frailty should cause frailty symptoms has not been explicitly declared in various, often conflicting, theories of frailty [2]. Causal relationships can be established through different approaches [9], such as using the Bradford-Hill Criteria [10]. Among all requirements in the Bradford-Hill Criteria, the strengths of associations between frailty and frailty symptoms are important and can be used to assess the impact of frailty prevention programs on frailty treatment and to understand the mechanisms that cause frailty. For example, it has been suggested that cognitive impairment plays an important role for frailty diagnosis and mortality among frail patients [3]. The confirmation of the causal relationship between frailty and cognitive function has the potential for intervention development. Without extensive reviews of the relationships between frailty and its symptoms, how frailty may influence frailty symptoms is an important question that is left unanswered. This study aims to assess the effect of frailty on the occurrence of frailty symptoms using a cohort that have been used to compare three of the most used frailty indices.

## Methods

The Health and Retirement Study (HRS) follows Americans aged 50 years and over every 2 years [1, 2, 11, 12]. The 2004 wave HRS data were used to compare frailty indices defined by 3 models: the Functional Domains Model by Strawbridge et al. [13], the Burden Model by Rockwood et al. [14, 15], and the Biologic Syndrome Model by Fried et al. [4]. Frailty symptoms or input variables were used to defined various domains defined by the 3 models [2, 3, 16]. When individuals presented enough numbers of frailty symptoms in a domain, these individuals might be considered to have a deficit in this domain for the Functional Domain Model and the Biologic Syndrome Model [2]. For example, the weight loss domain in the Biologic Syndrome Model asked individuals whether they had body mass index (BMI) less than 18.5 kg/m^2^ or whether they lost weight for 10% or more, compared to two years ago [1]. This domain required information on weights, heights, BMI, and weights two years ago [1].

In the Burden Model, one symptom represented a single domain and the presence of a symptom suggested the occurrence of a deficit [1, 2]. The frailty indices were the numbers of deficits identified using 4, 70, and 5 domains defined by the 3 frailty models, respectively [1]. Frailty statuses could be diagnosed when individuals had 2, 18 (70 times 0.25), or 3 deficits according to the 3 models, respectively [2]. The details of the frailty symptoms and input variables were published elsewhere (https://doi.org/10.1371/journal.pone.0197859.s002) [2]. The names and definitions of the input variables, frailty symptoms, and domains are listed in Tables 1–3. There were 10, 26, and 14 variables (frailty symptoms, input variables, or domains) identified for the 3 models, respectively. In total, there were 57 variables required to produce the frailty indices defined by the 3 models. In addition, there were 4 bias variables induced by the 4 domains in the Functional Domains Model (Table 1), 1 bias variable induced by the Burden Model (Table 2), and 4 bias variables induced by the Biologic Syndrome Model (Table 3) [2].

**Table 1:** Frailty status defined by the Functional Domains Model and its associations with frailty symptoms.

HRS variables	Definitions	N with symptoms for binomial variables	Odds ratios (95% CIs	Mean (SD)	Correlation coefficients (95% CIs)
**r7agey_b**	Age at interview (years)			73.92 (7.59)	0.26 (0.24 to 0.27)***
**r7bmi**	Self-reported body mass index = kg/m2			26.68 (5.31)	-0.02 (-0.04 to 0)
**r7cogtot**	Total cognition summary score			20.53 (6.08)	-0.41 (-0.42 to -0.39)***
**r7dizz**	Physical functioning: Dizziness as persistent problem	1577	7.9 (7.03 to 8.87)***	0.14 (0.35)	0.36 (0.35 to 0.38)***
**r7eye**	Sensory problems: Fair or poor eyesight despite use of corrective lenses			2.95 (1.01)	0.44 (0.42 to 0.45)***
**r7fall**	fallen down last 2 years			0.9 (2.88)	0.29 (0.27 to 0.31)***
**r7frailim1**	Frailty index: Functional Domains Model			0.98 (0.93)	0.85 (0.84 to 0.85)***
**r7frailim1cat**	Frailty status: Functional Domains Model (outcome of this table)	3059	Not applicable	0.28 (0.45)	1 (1 to 1)***
**r7hear**	Sensory problems: fair or poor hearing despite use of hearing aides			2.87 (1.12)	0.37 (0.35 to 0.39)***
**r7lift**	some difficulty in lift/carry 10lbs	3631	8.85 (8.05 to 9.72)***	0.33 (0.47)	0.46 (0.45 to 0.48)***
**r7memopr**	Proxy memory rating			1.39 (1.18)	0.33 (0.31 to 0.35)***
**r7wchange**	Weight in wave 2002 minus weight in wave 2004 (%)			0.01 (0.08)	-0.04 (-0.05 to -0.02)***
**ragender**	Male = 0; female = 1	6385	1.32 (1.21 to 1.44)***	0.57 (0.49)	0.06 (0.04 to 0.08)***
**Domains and other frailty symptoms identified by the other 2 models**					
**r7actsum**	Summary scores of physical activities			33.45 (9.11)	0.31 (0.29 to 0.33)***
**r7arthrcat**	Binomial: Arthritis	7693	1.84 (1.67 to 2.03)***	0.69 (0.46)	0.12 (0.1 to 0.14)***
**r7bathcat**	Binomial: Problems with bathing	1264	8.28 (7.27 to 9.42)***	0.11 (0.32)	0.34 (0.32 to 0.36)***
**r7cancrcat**	Binomial: Malignant disease	1978	1.03 (0.92 to 1.15)	0.18 (0.38)	0.01 (-0.01 to 0.02)
**r7cogimpair**	Impaired cognition based on performance-based scores or proxy assessment	999	23.09 (19.14 to 27.85)***	0.09 (0.29)	0.41 (0.4 to 0.43)***
**r7deprescat**	Binomial: Feeling sad, blue, depressed	2011	3.54 (3.2 to 3.91)***	0.18 (0.39)	0.24 (0.22 to 0.26)***
**r7diabscat**	Binomial: History of diabetes mellitus	292	1.38 (1.08 to 1.77)*	0.03 (0.16)	0.02 (0.01 to 0.04)**
**r7dresscat**	Binomial: problem getting dressed	1419	6.01 (5.34 to 6.76)***	0.13 (0.33)	0.31 (0.29 to 0.32)***
**r7effort**	everything an effort	2932	3.96 (3.62 to 4.34)***	0.26 (0.44)	0.29 (0.27 to 0.31)***
**r7fall_cat1**	More than 1 falls	3640	3.6 (3.3 to 3.93)***	0.33 (0.47)	0.28 (0.26 to 0.3)***
**r7fall_cat2**	More than 2 falls	1929	6.71 (6.04 to 7.46)***	0.17 (0.38)	0.36 (0.35 to 0.38)***
**r7frail1_1**	Dizziness as persistent problem, > = 2 falls in previous 2 years, or difficulty lifting 10 pounds	4383	27.81 (24.63 to 31.42)***	0.39 (0.49)	0.61 (0.6 to 0.62)***
**r7frail1_2**	Weight in wave 2002 minus weight in wave 2004! 10% of weight in wave 2002 or body mass index o18.5 kg/m2	866	9.35 (7.97 to 10.96)***	0.08 (0.27)	0.3 (0.29 to 0.32)***
**r7frail1_3**	Mild to severe cognitive impairment on performance-based measure or according to proxy and interviewer rating			0.09 (0.29)	0.42 (0.4 to 0.43)***
**r7frail1_4**	Fair or poor eyesight despite use of corrective lenses or fair or poor hearing despite use of hearing aides			0.42 (0.49)	0.59 (0.58 to 0.6)***
**r7frail3_2**	Yes to either of two CES-D items: (i) Felt that everything I did was an effort in last week. (ii) Could not get going in last week.	4025	3.99 (3.66 to 4.35)***	0.36 (0.48)	0.3 (0.29 to 0.32)***
**r7frail3_3**	Frequency of three intensities of activity, lowest quintile (stratified according to sex)	2872	4.11 (3.75 to 4.51)***	0.26 (0.44)	0.3 (0.28 to 0.32)***
**r7frail3_4**	Time to walk 8 ft, converted to time to walk 15 ft. Cutoff criteria according to sex and height remain the same	5474	1.44 (1.33 to 1.57)***	0.49 (0.5)	0.08 (0.06 to 0.1)***
**r7frail3_5**	Grip strength: Weakest 20% (stratified according to sex and BMI)	2529	1.43 (1.3 to 1.57)***	0.23 (0.42)	0.07 (0.05 to 0.09)***
**r7frailim1**	Frailty index: Functional Domains Model			0.98 (0.93)	0.85 (0.84 to 0.85)***
**r7frailim2**	Frailty index: Burden Model			5.02 (2.83)	0.46 (0.44 to 0.47)***
**r7frailim2cat**	Frailty status: Burden Model			0.45 (0.5)	0.38 (0.36 to 0.4)***
**r7frailim3**	Frailty index: Biologic Syndrome Model			1.14 (1.08)	0.35 (0.3 to 0.39)***
**r7frailim3cat**	Frailty status: Biologic Syndrome Model			0.12 (0.33)	0.3 (0.26 to 0.34)***
**r7going**	Could not get going	2682	3.41 (3.11 to 3.73)***	0.24 (0.43)	0.25 (0.24 to 0.27)***
**r7grip**	Grip strength, largest value			30.19 (16.32)	-0.07 (-0.09 to -0.06)***
**r7gripl**	Grip strength, left hand			26.63 (16.29)	-0.07 (-0.09 to -0.05)***
**r7gripr**	Grip strength, right hand			28.74 (16.14)	-0.1 (-0.12 to -0.08)***
**r7headac**	Headache	817	3.05 (2.64 to 3.53)***	0.07 (0.26)	0.15 (0.13 to 0.17)***
**r7heartcat**	Binomial: Cardiac problems	3631	2.15 (1.97 to 2.35)***	0.33 (0.47)	0.17 (0.15 to 0.18)***
**r7height**	Self-reported height in meters			1.68 (0.1)	-0.09 (-0.11 to -0.07)***
**r7hibp**	had high blood pressure since last interview	6791	1.49 (1.36 to 1.62)***	0.61 (0.49)	0.08 (0.07 to 0.1)***
**r7ltactx**	Frequency of light physical activity			2.87 (1.2)	0.29 (0.28 to 0.31)***
**r7lungcat**	Binomial: Lung problems	1345	2.02 (1.79 to 2.27)***	0.12 (0.33)	0.11 (0.09 to 0.13)***
**r7mdactx**	Frequency of moderate physical activity			3.13 (1.37)	0.29 (0.27 to 0.31)***
**r7memryscat**	Binomial: Memory changes	345	7.46 (5.86 to 9.48)***	0.03 (0.17)	0.18 (0.16 to 0.2)***
**r7mobila**	Some difficulty in mobility /05			1.36 (1.59)	0.43 (0.42 to 0.45)***
**r7muscle**	Musculoskeletal problems	386	1.3 (1.05 to 1.62)*	0.03 (0.18)	0.02 (0 to 0.04)*
**r7psychcat**	Binomial: Depression	1799	2.82 (2.55 to 3.13)***	0.16 (0.37)	0.19 (0.17 to 0.21)***
**r7psychscat**	Binomial: Changes in general mental functioning	246	3.6 (2.78 to 4.64)***	0.02 (0.15)	0.1 (0.08 to 0.12)***
**r7seizure**	Seizures, generalized	Not available			
**r7sleeprcat**	Binomial: Sleep changes	3102	2.26 (2.07 to 2.47)***	0.28 (0.45)	0.17 (0.15 to 0.19)***
**r7strokcat**	Binomial: Cerebrovascular problems	1139	3.36 (2.97 to 3.81)***	0.1 (0.3)	0.19 (0.17 to 0.21)***
**r7strokecat**	Binomial: History of stroke	1283	3.39 (3.01 to 3.82)***	0.12 (0.32)	0.2 (0.18 to 0.22)***
**r7stroks**	Had stroke since last interview	255	3.31 (2.58 to 4.25)***	0.02 (0.15)	0.09 (0.08 to 0.11)***
**r7tired**	Tiredness all the time	1	Not applicable	0 (0.01)	-0.01 (-0.02 to 0.01)
**r7toiltcat**	Binomial: Toileting problems	903	6.9 (5.95 to 8)***	0.08 (0.27)	0.27 (0.26 to 0.29)***
**r7underw**	Underweight in wave 2004 (%)	313	7.94 (6.15 to 10.25)***	0.03 (0.17)	0.18 (0.16 to 0.19)***
**r7urine**	Urinary incontinence	2710	2.35 (2.15 to 2.58)***	0.24 (0.43)	0.18 (0.16 to 0.19)***
**r7vgactx**	Frequency of vigorous physical activity			4.24 (1.24)	0.2 (0.18 to 0.22)***
**r7walkt**	Slowness: Time to walk 8 ft, converted to time to walk 15 ft. Cutoff criteria according to sex and height remain the same			5.09 (19.08)	0.11 (0.1 to 0.13)***
**r7walkt15**	Time to walk 15 feet			9.54 (35.77)	0.11 (0.1 to 0.13)***
**r7weight**	Self-reported weight in kilograms			76.03 (17.43)	-0.06 (-0.08 to -0.04)***
**Bias variables**					
**Biases induced by the Functional Domains Model**					
**r7frail1_1res**	Bias induced by Dizziness as persistent problem, > = 2 falls in previous 2 years, or difficulty lifting 10 pounds			0 (0.29)	0.35 (0.34 to 0.37)***
**r7frail1_2res**	Bias induced by Weight in wave 2002 minus weight in wave 2004! 10% of weight in wave 2002 or body mass index o18.5 kg/m2			0 (0.25)	0.2 (0.19 to 0.22)***
**r7frail1_3res**	Bias induced by Mild to severe cognitive impairment on performance-based measure or according to proxy and interviewer rating			0 (0.15)	-0.03 (-0.05 to -0.01)**
**r7frail1_4res**	Bias induced by Fair or poor eyesight despite use of corrective lenses or fair or poor hearing despite use of hearing aides			0 (0.2)	0.02 (0.01 to 0.04)*
**Biases induced by the Burden Model**					
**r7frail2res**	Bias induced by Summary of proxy memory rating and total cognition summary score			0 (0.04)	-0.14 (-0.16 to -0.11)***
**Biases induced by the Biologic Syndrome Model**					
**r7frail3_2res**	Bias induced by Yes to either of two CES-D items: (i) Felt that everything I did was an effort in last week. (ii) Could not get going in last week.			0 (0.17)	0.01 (-0.04 to 0.06)
**r7frail3_3res**	Bias induced by Frequency of three intensities of activity, lowest quintile (stratified according to sex)			0 (0.31)	0.1 (0.05 to 0.15)***
**r7frail3_4res**	Bias induced by Time to walk 8 ft, converted to time to walk 15 ft. Cutoff criteria according to sex and height remain the same			0 (0.49)	0.16 (0.12 to 0.21)***
**r7frail3_5res**	Bias induced by Grip strength: Weakest 20% (stratified according to sex and BMI)			0 (0.34)	0.05 (0 to 0.09)

n = 11,113; frailty n (%) = 3,059 (27.53%); mean age = 74.92 years; female % = 57.46%.

BMI = body mass index; CES-D = Center for Epidemiological Studies Depression; HRS = Health and Retirement Study.

* = p < 0.05

** = p < 0.01

*** = p < 0.001.

**Table 2:** Frailty status defined by the Burden Model and its associations with frailty symptoms.

HRS variables	Definitions	N with symptoms for binomial variables	Odds ratios (95% CIs	Mean (SD)	Correlation coefficients (95% CIs)
**r7agey_b**	Age at interview (years)			77.43 (6.46)	0.19 (0.17 to 0.22)***
**r7arthrcat**	Binomial: Arthritis	5457	4.45 (3.97 to 4.99)***	0.71 (0.45)	0.3 (0.28 to 0.32)***
**r7bathcat**	Binomial: Problems with bathing	1091	28.44 (21.95 to 36.86)***	0.14 (0.35)	0.4 (0.39 to 0.42)***
**r7cancrcat**	Binomial: Malignant disease	1489	2.12 (1.89 to 2.38)***	0.19 (0.39)	0.15 (0.13 to 0.17)***
**r7cogtot**	Total cognition summary score			19.69 (6.27)	-0.25 (-0.27 to -0.23)***
**r7deprescat**	Binomial: Feeling sad, blue, depressed	1480	5.55 (4.87 to 6.32)***	0.19 (0.39)	0.31 (0.29 to 0.33)***
**r7diabscat**	Binomial: History of diabetes mellitus	174	3.07 (2.21 to 4.26)***	0.02 (0.15)	0.08 (0.06 to 0.1)***
**r7dresscat**	Binomial: problem getting dressed	1159	18.97 (15.34 to 23.48)***	0.15 (0.36)	0.4 (0.38 to 0.42)***
**r7fall**	Fallen down last 2 years			0.99 (2.89)	0.3 (0.28 to 0.32)***
**r7frailim2**	Frailty index: Burden Model			5.02 (2.83)	0.8 (0.8 to 0.81)***
**r7frailim2cat**	Frailty status: Burden Model (outcome of this table)	3442	Not applicable	0.45 (0.5)	1 (1 to 1)***
**r7headac**	Headache	514	5.28 (4.25 to 6.58)***	0.07 (0.25)	0.19 (0.17 to 0.21)***
**r7heartcat**	Binomial: Cardiac problems	2817	4.01 (3.64 to 4.42)***	0.37 (0.48)	0.32 (0.3 to 0.34)***
**r7hibp**	Had high blood pressure since last interview	4809	2.81 (2.55 to 3.1)***	0.62 (0.48)	0.24 (0.22 to 0.26)***
**r7lungcat**	Binomial: Lung problems	928	4.41 (3.77 to 5.16)***	0.12 (0.33)	0.23 (0.2 to 0.25)***
**r7memopr**	Proxy memory rating			1.45 (1.29)	0.23 (0.21 to 0.25)***
**r7memryscat**	Binomial: Memory changes	303	5.95 (4.43 to 8)***	0.04 (0.19)	0.15 (0.13 to 0.17)***
**r7mobila**	Some difficulty in mobility /05			1.51 (1.66)	0.56 (0.55 to 0.58)***
**r7muscle**	Musculoskeletal problems	258	3.61 (2.73 to 4.78)***	0.03 (0.18)	0.11 (0.09 to 0.13)***
**r7psychcat**	Binomial: Depression	1230	7.74 (6.63 to 9.04)***	0.16 (0.37)	0.33 (0.31 to 0.35)***
**r7psychscat**	Binomial: Changes in general mental functioning	188	15.02 (8.84 to 25.5)***	0.02 (0.15)	0.15 (0.13 to 0.17)***
**r7seizure**	Seizures, generalized	0	Not applicable	0	Not applicable for uniform values
**r7sleeprcat**	Binomial: Sleep changes	2185	5.05 (4.53 to 5.63)***	0.28 (0.45)	0.35 (0.33 to 0.37)***
**r7strokcat**	Binomial: Cerebrovascular problems	923	11.45 (9.34 to 14.03)***	0.12 (0.32)	0.32 (0.3 to 0.34)***
**r7strokecat**	Binomial: History of stroke	1062	10.42 (8.68 to 12.51)***	0.14 (0.34)	0.34 (0.32 to 0.36)***
**r7tired**	Tiredness all the time	1	Not applicable	0 (0.01)	0.01 (-0.01 to 0.03)
**r7toiltcat**	Binomial: Toileting problems	759	44.18 (29.79 to 65.52)***	0.1 (0.3)	0.35 (0.33 to 0.36)***
**r7urine**	Urinary incontinence	2075	6.41 (5.71 to 7.19)***	0.27 (0.44)	0.38 (0.36 to 0.4)***
**ragender**	Male = 0; female = 1	4534	1.69 (1.54 to 1.85)***	0.59 (0.49)	0.13 (0.1 to 0.15)***
**Domains and other frailty symptoms identified by the other 2 models**					
**r7actsum**	Summary scores of physical activities			34.44 (8.92)	0.35 (0.33 to 0.37)***
**r7bmi**	Self-reported body mass index = kg/m2			26.1 (5.07)	0.07 (0.05 to 0.1)***
**r7cogimpair**	Impaired cognition based on performance-based scores or proxy assessment	884	4.71 (4 to 5.54)***	0.11 (0.32)	0.23 (0.21 to 0.25)***
**r7dizz**	Physical functioning: Dizziness as persistent problem	1163	3.93 (3.42 to 4.5)***	0.15 (0.36)	0.23 (0.21 to 0.25)***
**r7effort**	Everything an effort	2153	4.59 (4.12 to 5.11)***	0.28 (0.45)	0.33 (0.31 to 0.35)***
**r7eye**	Sensory problems: Fair or poor eyesight despite use of corrective lenses			3 (1.03)	0.26 (0.24 to 0.28)***
**r7fall_cat1**	More than 1 falls	2761	5.61 (5.06 to 6.21)***	0.36 (0.48)	0.39 (0.37 to 0.41)***
**r7fall_cat2**	More than 2 falls	1499	6.94 (6.06 to 7.95)***	0.19 (0.4)	0.35 (0.33 to 0.37)***
**r7frail1_1**	Dizziness as persistent problem, > = 2 falls in previous 2 years, or difficulty lifting 10 pounds	3290	6.02 (5.45 to 6.64)***	0.43 (0.49)	0.42 (0.4 to 0.44)***
**r7frail1_2**	Weight in wave 2002 minus weight in wave 2004! 10% of weight in wave 2002 or body mass index o18.5 kg/m2	635	1.97 (1.67 to 2.32)***	0.08 (0.27)	0.09 (0.07 to 0.11)***
**r7frail1_3**	Mild to severe cognitive impairment on performance-based measure or according to proxy and interviewer rating			0.11 (0.32)	0.23 (0.21 to 0.25)***
**r7frail1_4**	Fair or poor eyesight despite use of corrective lenses or fair or poor hearing despite use of hearing aides			0.45 (0.5)	0.22 (0.2 to 0.24)***
**r7frail3_2**	Yes to either of two CES-D items: (i) Felt that everything I did was an effort in last week. (ii) Could not get going in last week.	2977	4.72 (4.28 to 5.21)***	0.39 (0.49)	0.36 (0.34 to 0.38)***
**r7frail3_3**	Frequency of three intensities of activity, lowest quintile (stratified according to sex)	2226	4.89 (4.39 to 5.45)***	0.29 (0.45)	0.34 (0.32 to 0.36)***
**r7frail3_4**	Time to walk 8 ft, converted to time to walk 15 ft. Cutoff criteria according to sex and height remain the same	3853	1.37 (1.25 to 1.5)***	0.5 (0.5)	0.08 (0.06 to 0.1)***
**r7frail3_5**	Grip strength: Weakest 20% (stratified according to sex and BMI)	1840	1.4 (1.26 to 1.55)***	0.24 (0.43)	0.07 (0.05 to 0.09)***
**r7frailim1**	Frailty index: Functional Domains Model			1.08 (0.96)	0.43 (0.41 to 0.45)***
**r7frailim1cat**	Frailty status: Functional Domains Model	2434	5.57 (5.01 to 6.19)***	0.32 (0.46)	0.38 (0.36 to 0.4)***
**r7frailim2**	Frailty index: Burden Model			5.02 (2.83)	0.8 (0.8 to 0.81)***
**r7frailim3**	Frailty index: Biologic Syndrome Model			1.22 (1.11)	0.28 (0.23 to 0.33)***
**r7frailim3cat**	Frailty status: Biologic Syndrome Model			0.14 (0.35)	0.19 (0.14 to 0.24)***
**r7going**	could not get going	2026	4.64 (4.15 to 5.18)***	0.26 (0.44)	0.32 (0.3 to 0.34)***
**r7grip**	Grip strength, largest value			29.49 (15.93)	-0.08 (-0.1 to -0.06)***
**r7gripl**	Grip strength, left hand			25.89 (15.72)	-0.07 (-0.09 to -0.04)***
**r7gripr**	Grip strength, right hand			27.99 (15.37)	-0.11 (-0.13 to -0.09)***
**r7hear**	Sensory problems: fair or poor hearing despite use of hearing aides			2.97 (1.13)	0.16 (0.14 to 0.18)***
**r7height**	Self-reported height in meters			1.68 (0.1)	-0.1 (-0.13 to -0.08)***
**r7lift**	Some difficulty in lift/carry 10lbs	2862	5.88 (5.31 to 6.51)***	0.37 (0.48)	0.4 (0.39 to 0.42)***
**r7ltactx**	Frequency of light physical activity			2.99 (1.24)	0.3 (0.28 to 0.32)***
**r7mdactx**	Frequency of moderate physical activity			3.24 (1.41)	0.34 (0.32 to 0.36)***
**r7stroks**	Had stroke since last interview	208	13 (8.09 to 20.88)***	0.03 (0.16)	0.15 (0.13 to 0.18)***
**r7underw**	Underweight in wave 2004 (%)	270	1.68 (1.31 to 2.14)***	0.04 (0.18)	0.05 (0.03 to 0.07)***
**r7vgactx**	Frequency of vigorous physical activity			4.35 (1.19)	0.23 (0.21 to 0.25)***
**r7walkt**	Slowness: Time to walk 8 ft, converted to time to walk 15 ft. Cutoff criteria according to sex and height remain the same			5.2 (19.49)	0.12 (0.1 to 0.15)***
**r7walkt15**	Time to walk 15 feet			9.75 (36.55)	0.12 (0.1 to 0.15)***
**r7wchange**	Weight in wave 2002 minus weight in wave 2004 (%)			0.01 (0.08)	0.04 (0.02 to 0.07)***
**r7weight**	Self-reported weight in kilograms			73.91 (16.73)	0 (-0.02 to 0.03)
**Bias variables**					
**Bias variables induced by the Functional Domains Model**					
**r7frail1_1res**	Bias induced by Dizziness as persistent problem, > = 2 falls in previous 2 years, or difficulty lifting 10 pounds			0.01 (0.3)	0.19 (0.17 to 0.21)***
**r7frail1_2res**	Bias induced by Weight in wave 2002 minus weight in wave 2004! 10% of weight in wave 2002 or body mass index o18.5 kg/m2			0 (0.26)	0.15 (0.13 to 0.17)***
**r7frail1_3res**	Bias induced by Mild to severe cognitive impairment on performance-based measure or according to proxy and interviewer rating			0 (0.17)	-0.01 (-0.03 to 0.01)
**r7frail1_4res**	Bias induced by Fair or poor eyesight despite use of corrective lenses or fair or poor hearing despite use of hearing aides			0 (0.21)	0.01 (-0.01 to 0.03)
**Bias variables induced by the Burden Model**					
**r7frail2res**	Bias induced by Summary of proxy memory rating and total cognition summary score			0 (0.04)	-0.06 (-0.08 to -0.04)***
**Bias variables induced by the Biologic Syndrome Model**					
**r7frail3_2res**	Bias induced by Yes to either of two CES-D items: (i) Felt that everything I did was an effort in last week. (ii) Could not get going in last week.			0 (0.17)	-0.04 (-0.09 to 0.01)
**r7frail3_3res**	Bias induced by Frequency of three intensities of activity, lowest quintile (stratified according to sex)			0 (0.32)	0.12 (0.07 to 0.17)***
**r7frail3_4res**	Bias induced by Time to walk 8 ft, converted to time to walk 15 ft. Cutoff criteria according to sex and height remain the same			0.03 (0.49)	0.09 (0.03 to 0.14)**
**r7frail3_5res**	Bias induced by Grip strength: Weakest 20% (stratified according to sex and BMI)			0.01 (0.35)	0.03 (-0.03 to 0.08)

n = 7,713; frailty n (%) = 6,755 (87.58%); mean age = 78.43 years; female % = 58.78%.

BMI = body mass index; CES-D = Center for Epidemiological Studies Depression; HRS = Health and Retirement Study.

* = p < 0.05

** = p < 0.01

*** = p < 0.001.

**Table 3:** Frailty status defined by the Biologic Syndrome Model and its associations with frailty symptoms.

HRS	Definitions variables	N with symptoms for binomial variables	Odds ratios (95% CIs	Mean (SD)	Correlation coefficients (95% CIs)
**r7agey_b**	Age at interview (years)			76.05 (7.36)	0.23 (0.19 to 0.28)***
**r7bmi**	Self-reported body mass index = kg/m2			26.41 (4.91)	-0.04 (-0.09 to 0.01)
**r7cogtot**	Total cognition summary score			21.44 (4.79)	-0.22 (-0.26 to -0.17)***
**r7effort**	Everything an effort	258	5.56 (4.04 to 7.66)***	0.16 (0.36)	0.28 (0.23 to 0.32)***
**r7frailim3**	Frailty index: Biologic Syndrome Model			1.14 (1.08)	0.73 (0.7 to 0.75)***
**r7frailim3cat**	Frailty status: Biologic Syndrome Model (outcome of this table)	203	Not applicable	0.12 (0.33)	1 (1 to 1)***
**r7going**	Could not get going	306	6.43 (4.7 to 8.8)***	0.19 (0.39)	0.31 (0.27 to 0.36)***
**r7gripl**	Grip strength, left hand			24.85 (13.1)	-0.28 (-0.32 to -0.23)***
**r7gripr**	Grip strength, right hand			27.38 (13.46)	-0.32 (-0.36 to -0.28)***
**r7height**	Self-reported height in meters			1.69 (0.1)	-0.12 (-0.17 to -0.08)***
**r7mdactx**	Frequency of moderate physical activity			2.93 (1.3)	0.41 (0.37 to 0.45)***
**r7memopr**	Proxy memory rating (1 to 6)			1 (0)	Not applicable for uniform values
**r7stroks**	had stroke since last interview (1 = no, 2 = yes)			1 (0)	Not applicable for uniform values
**r7vgactx**	Frequency of vigorous physical activity			4.18 (1.26)	0.21 (0.16 to 0.26)***
**r7walkt**	Slowness: Time to walk 8 ft, converted to time to walk 15 ft. Cutoff criteria according to sex and height remain the same			4.14 (13.29)	0.42 (0.38 to 0.46)***
**ragender**	Male = 0; female = 1	898	2.61 (1.88 to 3.64)***	0.55 (0.5)	0.14 (0.1 to 0.19)***
**Domains and frailty symptoms identified by the other 2 models**					
**r7actsum**	Summary scores of physical activities			32.41 (8.61)	0.37 (0.33 to 0.41)***
**r7arthrcat**	Binomial: Arthritis	1140	2.4 (1.63 to 3.52)***	0.69 (0.46)	0.11 (0.06 to 0.16)***
**r7bathcat**	Binomial: Problems with bathing	88	6.75 (4.3 to 10.59)***	0.05 (0.23)	0.23 (0.18 to 0.28)***
**r7cancrcat**	Binomial: Malignant disease	300	1.28 (0.89 to 1.83)	0.18 (0.39)	0.03 (-0.02 to 0.08)
**r7cogimpair**	Impaired cognition based on performance-based scores or proxy assessment	45	5.09 (2.75 to 9.42)***	0.03 (0.16)	0.14 (0.09 to 0.19)***
**r7deprescat**	Binomial: Feeling sad, blue, depressed	0	Not applicable	0	Not applicable for uniform values
**r7diabscat**	Binomial: History of diabetes mellitus	42	1.7 (0.77 to 3.72)	0.03 (0.16)	0.03 (-0.02 to 0.08)
**r7dizz**	Physical functioning: Dizziness as persistent problem	176	2 (1.34 to 2.98)**	0.11 (0.31)	0.09 (0.04 to 0.13)***
**r7dresscat**	Binomial: problem getting dressed	120	3.64 (2.39 to 5.54)***	0.07 (0.26)	0.16 (0.11 to 0.2)***
**r7eye**	Sensory problems: Fair or poor eyesight despite use of corrective lenses			2.85 (0.97)	0.13 (0.09 to 0.18)***
**r7fall**	fallen down last 2 years			0.75 (2.34)	0.11 (0.06 to 0.16)***
**r7fall_cat1**	More than 1 falls	513	2.2 (1.63 to 2.96)***	0.31 (0.46)	0.13 (0.08 to 0.18)***
**r7fall_cat2**	More than 2 falls	242	2.34 (1.65 to 3.31)***	0.15 (0.35)	0.12 (0.07 to 0.17)***
**r7frail1_1**	Dizziness as persistent problem, > = 2 falls in previous 2 years, or difficulty lifting 10 pounds	573	3.3 (2.44 to 4.46)***	0.35 (0.48)	0.2 (0.15 to 0.24)***
**r7frail1_2**	Weight in wave 2002 minus weight in wave 2004! 10% of weight in wave 2002 or body mass index o18.5 kg/m2	94	8.49 (5.49 to 13.14)***	0.06 (0.23)	0.27 (0.23 to 0.32)***
**r7frail1_3**	Mild to severe cognitive impairment on performance-based measure or according to proxy and interviewer rating	45	5.09 (2.75 to 9.42)***	0.03 (0.16)	0.14 (0.09 to 0.19)***
**r7frail1_4**	Fair or poor eyesight despite use of corrective lenses or fair or poor hearing despite use of hearing aides	645	2.24 (1.66 to 3.02)***	0.39 (0.49)	0.13 (0.09 to 0.18)***
**r7frail3_2**	Yes to either of two CES-D items: (i) Felt that everything I did was an effort in last week. (ii) Could not get going in last week.	441	9.93 (7.13 to 13.84)***	0.27 (0.44)	0.38 (0.34 to 0.42)***
**r7frail3_3**	Frequency of three intensities of activity, lowest quintile (stratified according to sex)	329	17.58 (12.47 to 24.78)***	0.2 (0.4)	0.49 (0.45 to 0.52)***
**r7frail3_4**	Time to walk 8 ft, converted to time to walk 15 ft. Cutoff criteria according to sex and height remain the same	724	27.06 (14.95 to 48.96)***	0.44 (0.5)	0.38 (0.34 to 0.42)***
**r7frail3_5**	Grip strength: Weakest 20% (stratified according to sex and BMI)	290	12.72 (9.18 to 17.64)***	0.18 (0.38)	0.44 (0.4 to 0.48)***
**r7frailim1**	Frailty index: Functional Domains Model			0.83 (0.8)	0.31 (0.26 to 0.35)***
**r7frailim1cat**	Frailty status: Functional Domains Model	340	5.91 (4.34 to 8.06)***	0.21 (0.41)	0.3 (0.26 to 0.34)***
**r7frailim2**	Frailty index: Burden Model			4.17 (2.07)	0.27 (0.22 to 0.32)***
**r7frailim2cat**	Frailty status: Burden Model			0.33 (0.47)	0.19 (0.14 to 0.24)***
**r7frailim3**	Frailty index: Biologic Syndrome Model			1.14 (1.08)	0.73 (0.7 to 0.75)***
**r7grip**	Grip strength, largest value			28.31 (13.69)	-0.32 (-0.36 to -0.27)***
**r7headac**	Headache	74	1.11 (0.56 to 2.2)	0.05 (0.21)	0.01 (-0.04 to 0.06)
**r7hear**	Sensory problems: fair or poor hearing despite use of hearing aides			2.88 (1.09)	0.11 (0.06 to 0.16)***
**r7heartcat**	Binomial: Cardiac problems	562	1.83 (1.36 to 2.46)***	0.34 (0.47)	0.1 (0.05 to 0.15)***
**r7hibp**	Had high blood pressure since last interview	987	1.51 (1.11 to 2.07)**	0.6 (0.49)	0.06 (0.02 to 0.11)**
**r7lift**	Some difficulty in lift/carry 10lbs	440	6.83 (4.98 to 9.35)***	0.27 (0.44)	0.32 (0.28 to 0.37)***
**r7ltactx**	Frequency of light physical activity			2.71 (1.06)	0.25 (0.2 to 0.29)***
**r7lungcat**	Binomial: Lung problems	165	1.84 (1.21 to 2.79)**	0.1 (0.3)	0.07 (0.02 to 0.12)**
**r7memryscat**	Binomial: Memory changes	20	1.79 (0.59 to 5.4)	0.01 (0.11)	0.03 (-0.02 to 0.07)
**r7mobila**	Some difficulty in mobility /05			1.12 (1.38)	0.37 (0.33 to 0.41)***
**r7muscle**	Musculoskeletal problems	57	0.53 (0.19 to 1.47)	0.03 (0.18)	-0.03 (-0.08 to 0.02)
**r7psychcat**	Binomial: Depression	165	2.38 (1.6 to 3.54)***	0.1 (0.3)	0.11 (0.06 to 0.16)***
**r7psychscat**	Binomial: Changes in general mental functioning	17	1.53 (0.43 to 5.36)	0.01 (0.1)	0.02 (-0.03 to 0.06)
**r7seizure**	Seizures, generalized	0	Not applicable		Not applicable for uniform values
**r7sleeprcat**	Binomial: Sleep changes	346	1.74 (1.25 to 2.41)**	0.21 (0.41)	0.08 (0.03 to 0.13)***
**r7strokcat**	Binomial: Cerebrovascular problems	110	2.65 (1.68 to 4.18)***	0.07 (0.25)	0.11 (0.06 to 0.15)***
**r7strokecat**	Binomial: History of stroke	139	2.56 (1.68 to 3.88)***	0.08 (0.28)	0.11 (0.06 to 0.16)***
**r7tired**	Tiredness all the time	0	Not applicable		Not applicable for uniform values
**r7toiltcat**	Binomial: Toileting problems	60	6.07 (3.56 to 10.35)***	0.04 (0.19)	0.18 (0.14 to 0.23)***
**r7underw**	Underweight in wave 2004 (%)	36	9.72 (4.95 to 19.09)***	0.02 (0.15)	0.2 (0.15 to 0.24)***
**r7urine**	Urinary incontinence	360	2.14 (1.56 to 2.94)***	0.22 (0.41)	0.12 (0.07 to 0.17)***
**r7walkt15**	Time to walk 15 feet			7.76 (24.91)	0.42 (0.38 to 0.46)***
**r7wchange**	Weight in wave 2002 minus weight in wave 2004 (%)			0 (0.06)	0.05 (0 to 0.1)*
**r7weight**	Self-reported weight in kilograms			75.75 (16.87)	-0.1 (-0.15 to -0.05)***
**Bias variables**					
**Bias variables induced by the Functional Domains Model**					
**r7frail1_1res**	Bias induced by Dizziness as persistent problem, > = 2 falls in previous 2 years, or difficulty lifting 10 pounds			0 (0.29)	0.03 (-0.02 to 0.08)
**r7frail1_2res**	Bias induced by Weight in wave 2002 minus weight in wave 2004! 10% of weight in wave 2002 or body mass index o18.5 kg/m2			-0.03 (0.22)	0.21 (0.17 to 0.26)***
**r7frail1_3res**	Bias induced by Mild to severe cognitive impairment on performance-based measure or according to proxy and interviewer rating			0 (0.16)	-0.02 (-0.06 to 0.03)
**r7frail1_4res**	Bias induced by Fair or poor eyesight despite use of corrective lenses or fair or poor hearing despite use of hearing aides			0 (0.21)	0 (-0.05 to 0.05)
**Bias variables induced by the Burden Model**					
**r7frail2res**	Bias induced by Summary of proxy memory rating and total cognition summary score			0 (0.01)	-0.21 (-0.27 to -0.16)***
**Bias variables induced by the Biologic Syndrome Model**					
**r7frail3_2res**	Bias induced by Yes to either of two CES-D items: (i) Felt that everything I did was an effort in last week. (ii) Could not get going in last week.			0 (0.17)	0.08 (0.03 to 0.13)***
**r7frail3_3res**	Bias induced by Frequency of three intensities of activity, lowest quintile (stratified according to sex)			0 (0.31)	0.34 (0.3 to 0.38)***
**r7frail3_4res**	Bias induced by Time to walk 8 ft, converted to time to walk 15 ft. Cutoff criteria according to sex and height remain the same			0 (0.49)	0.3 (0.26 to 0.34)***
**r7frail3_5res**	Bias induced by Grip strength: Weakest 20% (stratified according to sex and BMI)			0 (0.34)	0.26 (0.22 to 0.31)***

n = 1,642; frailty n (%) = 540 (32.89%); mean age = 77.05 years; female % = 54.69s%. HRS = Health and Retirement Study.

BMI = body mass index; CES-D = Center for Epidemiological Studies Depression; HRS = Health and Retirement Study.

* = p < 0.05

** = p < 0.01

*** = p < 0.001.

### Statistical analyses

The associations between the frailty statuses and their frailty symptoms were determined with odds ratios and correlation coefficients. Odds ratios were the ratios of the odds of developing symptoms occurred among frail individuals, compared to the odds among those not frail [17]. Odds ratios were applicable to binomial variables [17]. Odds ratios equaling 1 suggest that the two groups have similar risks of developing symptoms [17]. The processing to transform non-binomial variables to binomial variables were according to the recommendations by the authors of the Burden Model [18]. Pearson’s correlation coefficients were used to assess the associations between frailty statuses defined by the 3 models and frailty symptoms or input variables or domains or bias variables [19]. Correlation coefficients ranged from -1 to 1, representing completely opposite information and identical information between 2 variables, respectively. We hypothesized that 1) frailty statuses were not associated with symptom incidence (odds ratio = 1); 2) frailty statuses were not correlated with frailty symptoms or input variables of the frailty indices (correlation coefficient = 0). Correlation coefficients between 0 and 0.10, 0.10 and 0.39, 0.40 and 0.69, 0.70 and 0.89, and 0.90 and 1.00 were interpreted as negligible, weak, moderate, strong, and very strong correlations, respectively [20]. P values were adjusted for multiple comparison using false discovery rates [21]. Two-tailed P values that were less than 0.05 were considered statistically significant. All statistical analyses were conducted within R environment (v4.0.4) [22] and RStudio (v1.4.1106) [23]. This secondary data analysis was approved by the ethics review committee at the Centre Hospitalier de l’Université de Montréal.

## Results

There were 11,113, 7,713, and 1,642 HRS participants analyzed for the frailty indices defined by the Functional Domains Model, the Burden Model, and the Biologic Syndrome Model in Tables 1–3, respectively [2]. The numbers of frail patients were 3,059 (27.53%), 3,442 (44.63%), and 203 (12.36%), respectively [2]. The mean ages were 74.92, 78.43, and 77.05 years, respectively. The proportions of females were 57.46%, 58.78%, and 54.69%, respectively.

### Frailty symptom development based on frailty status

In Tables 1–3, the associations between frailty status (yes or no) and symptom development are shown using odds ratios and correlation coefficients. Overall, most of frailty symptoms were significantly associated with frailty statuses. However, frailty statuses defined by the three models were not significantly associated with all frailty symptoms or input variables or domains. The frailty symptoms or input variables or domains that were not significantly associated with frailty statuses are described below. The correlation coefficients between the three frailty statuses ranged from 0.19 to 0.38 (weak correlations, p < 0.001 for all).

In Table 1, the frailty status defined by the Functional Domains Model was not significantly correlated with one input variable, BMI (correlation coefficients = -0.02, 95% CI = -0.04 to 0).

Among the frailty symptoms or input variables or domains identified by the other two models, two symptoms, malignant disease and tiredness all the time, were not significantly associated with this frailty status (p of correlations > 0.05 for both symptoms). Among the bias variables, one bias variable that was induced by having one of two Center for Epidemiologic Studies Depression (CES-D) items was not significantly associated with this frailty status (correlation p > 0.05).

In Table 2, the frailty status defined by the Burden Model was assessed for the associations with frailty symptoms, input variables, and domains. One input symptom, tiredness all the time, was not significantly associated with this frailty status (p of correlation > 0.05). One symptom identified by the other two models, self-reported weight, was not significantly correlated with this frailty status (p > 0.05). Among the bias variables, four were not significantly correlated with this frailty status (p > 0.05 for all).

In Table 3, the frailty status defined by the Biologic Syndrome Model was assessed for the associations with frailty symptoms, input variables, and domains. BMI was not significantly correlated with this frailty status (p > 0.05). Because the values of two symptoms, proxy memory rating and history of stroke, were the same for frail and non-frail HRS participants for this frailty index, their correlations with this frailty status could not be assessed. Six symptoms defined by the other two models, history of malignant disease, diabetes mellitus, headache, memory change, musculoskeletal problems, and change in general mental functioning, were not significantly correlated with this frailty status (p > 0.05 for all).

### Correlations with bias variables

In Tables 1–3, the correlations with bias variables are shown for the three frailty indices. Each frailty status was significantly associated with the bias variables induced by their own diagnostic criteria. The frailty statuses defined by the Functional Domains Model, the Burden Model, and the Biologic Syndrome Model, were significantly correlated with four, one, and four bias variables induced by their own models, respectively (p < 0.05 for all). In addition, the frailty status defined by the Functional Domains Model, the Burden Model, and the Biologic Syndrome Model, were significantly correlated with three, four, and two bias variables induced by the other two models, respectively (p < 0.05 for all).

## Discussion

Strengths of the associations are one of the Bradford-Hill criteria to assess whether a disease causes symptoms or outcomes [10]. Frailty has been promising in establishing causal relationships with major health outcomes, such as mortality and falls, based on frailty’s significant associations with them [2]. However, whether frailty should cause frailty symptoms has not been declared in the theories of frailty and whether frailty causes frailty symptoms have not been well studied. In this study using the HRS data, three of the most used frailty diagnoses fail to demonstrate significant correlations with some of the frailty symptoms of their own or those defined by the other two frailty diagnoses. When frailty lacks significant associations with frailty symptoms, this suggests frailty diagnoses are made based on so-called frailty symptoms, some of which frailty may not cause them. This needs serious discussion and examination.

### Why frailty fails to be significantly associated with frailty symptoms?

Frailty diagnoses do not fully support the models of their own by showing insignificant correlations with some of their own frailty symptoms or input variables. One reason may be that frailty researchers did not recognize the importance of causation between frailty and frailty symptoms. The authors of the Burden Model recommended selecting frailty symptoms based on the associations between the candidate symptoms and two factors: age and general health status [18], rather than selecting the symptoms that cause or are caused by frailty. For this model, the so-called frailty symptoms are, in fact, age-related and general health-related variables.

Second, for the Burden Model that requires a large number of frailty symptoms [18], some symptoms may not present in the population at all and are used for frailty diagnosis regardless. For example, in the HRS cohort, since we did not identify any patients with generalized seizures and it was impossible to determine the association between this symptom and the frailty status defined by this model. Including frailty symptoms that do not present in a population can underestimate the prevalence rate of frailty defined by the Burden Model. This is because the diagnostic threshold of this model is proportional to the total number of frailty symptoms used [18]. When a symptom that should not be included in the diagnostic criteria is included, the diagnostic threshold increases with the total number of frailty symptoms. If the symptom, generalized seizures, is excluded from the diagnostic criteria, the diagnostic threshold can decrease and it is likely that more HRS participants can qualify the diagnosis of frailty defined by the Burden Model.

The third reason is that the diagnostic criteria of frailty have been made so complicated that biases have been introduced and interfered the relationships between frailty and frailty symptoms. The biases are produced by censoring the sum of multiple symptoms and dichotomizing continuous variables [2]. These biases are so important that each of the frailty indices defined by the three models is significantly associated with the biases created by their own or the other two models. In fact, the biases generated by the diagnostic criteria of the Biologic Syndrome Model explain this model’s frailty index better than its frailty symptoms [2]. When the frailty indices better represent the biases, these indices are less likely to have significant associations with their frailty symptoms.

The last reason is the correlations between frailty symptoms have been neglected. The correlations between symptoms, symptom prevalence, and the design of the diagnostic criteria (whether biases are created and integrated to the diagnosis) are the three major determinants of the prevalence of the diagnosis [24]. Neglecting the importance of symptom correlations can lead to major errors. For example, when five highly correlated variables with the same means (correlation coefficients = 1) are summed to create an index, this index is not very different from five times any one of the five input variables [25]. When two completely opposite variables with mean values of 0 (correlation coefficient = -1) are summed, the derived index contains only 0 [25]. The frailty indices defined by the three frailty models consist of frailty symptoms of various correlations with each other. Some of the frailty symptoms in the three models may be highly correlated with each other. It takes only four frailty symptoms to explain more than 54% of the variances of the three frailty indices that use 9 or more frailty symptoms [2]. This issue becomes more problematic for frailty diagnoses made based on a large number of frailty symptoms. The frailty status defined by the Burden Model requires at least 30 symptoms for diagnosis and 70 symptoms have often been used [18]. It took only 11 frailty symptoms to explain more than 90% of the variances of the frailty index [2]. The frailty status defined by this model is not significantly associated with two of its frailty symptoms in this study. The role of insignificant symptoms, seizure and tiredness, in the frailty status defined by the Burden Model haven’t been discussed in previous studies [18].

### Causation?

In addition to the strengths of associations, the evidence to support the causal relationship between frailty and frailty symptoms seems limited. The pathological changes that are considered related to frailty include sarcopenia, heart disease, and lung disease, depending on the frailty models [2]. In this study, grip strength, cardiac problems, and lung problems were significantly correlated with the three frailty indices, respectively. However, the biological and pathological evidence that support the causal relationship between frailty and the frailty symptoms of the other organ systems seems insufficient [26].

### Assumptions

The different patterns of the insignificance between frailty symptoms and the 3 frailty statuses indicated conflicting views on frailty. The 3 frailty models have major discrepancies in the underlying assumptions, including theoretical frameworks, age thresholds, the selection of frailty symptoms, and the design of diagnostic criteria [2]. Subsequently, the differences in these assumptions between frailty models can be shown with the symptoms that best explained frailty statuses [2]. We found the frailty symptoms or input variables that had the largest correlation coefficients with the three frailty statuses were different. The three symptoms that have the largest significant correlation coefficients with the frailty indices defined by the Functional Domain Model, the Burden Model, and the Biologic Syndrome Model are some difficulties in lifting 10 pounds, some difficulty in mobility, and slowness measured by time to walk 8 feet, respectively. The paths from non-frailty to frailty vary depending on the models used.

### Logic challenges

These results highlight logic challenges. Frail patients are not more likely to have certain frailty symptoms, but these symptoms are necessary to make these diagnoses. It is unclear whether the symptoms that frailty is insignificantly correlated with can be called “frailty” symptom or used for frailty diagnosis. When excluding these symptoms from being used for the diagnosis of frailty, the prevalence of frailty will likely decrease for the Functional Domain Model and the Biologic Syndrome Model and may increase or decrease for the Burden Model. Whether the updated indices will become insignificantly associated with other frailty symptoms is unclear. If the frailty symptoms that are not significantly associated with frailty should be excluded, many of the published frailty prevalence rates are likely to be overestimated or biased for the reason describe above. We have not identified any studies explicitly examine the significance of the associations between the frailty statuses and frailty symptoms they defined in their own models. We will continue exploring the causal relationship between frailty and frailty symptoms using other data sets in the future.

### Limitations

This study has strengths in using a publicly accessible database that has been investigated in previous studies [1, 2]. The demographic characteristics reported in this study matched those reported [1]. However, there are several limitations to this study. There are other statistical and epidemiological measures of association that can be tested to demonstrate the strengths of associations, including Chi-squared statistics and risk ratios [27, 28]. Odds ratios are adequate for cross-sectional studies to approximate risk ratios [17]. However, odds ratios can over- or under-estimate effect sizes if the underlying risk ratios are greater or less than 1, respectively [29]. Other measures of association will be explored in the future. Moreover, there are other factors influencing the correlations between frailty statuses and frailty symptoms, such as demographic characteristics. These factors can be adjusted using techniques, such as multiple regression [30, 31]. Lastly, this study used cross-sectional data and longitudinal follow-up of the strengths of the associations between frailty and frailty symptoms might help to answer important questions, such as whether the insignificant associations are transient, whether frailty predicts major outcomes better if insignificant frailty symptoms are discarded, and whether the biases induced by the frailty diagnostic criteria predict outcomes better than frailty symptoms. This will need to be explored in future research.

## Conclusion

The frailty diagnoses defined by three models were assessed for their correlations with frailty symptoms of their own, those defined by the other two models, and bias variables using odds ratios and correlation coefficients. Frailty diagnoses lack significant correlations with some of their own frailty symptoms and some of the frailty symptoms defined by the other two models. This suggests that frail patients are not more likely to have certain frailty symptoms using any of the three frailty models. This finding raises questions like whether frailty symptoms lacking significant correlations with frailty statuses could be included to diagnose frailty and whether frailty exists and causes frailty symptoms. Further research to assess the causal relationships between frailty and frailty symptoms is needed and planned.
