# Supplementary material for: Frailty does not cause all frail symptoms: United States Health and Retirement Study
Source: PLoS One. 2022 Nov 2;17(11):e0272289. doi: 10.1371/journal.pone.0272289 (PMC9629634; doi:10.1371/journal.pone.0272289)
Supplement: Attachment — Submitted filename: PONE-D-21-33729_FC.pdf [file pone.0272289.s002.pdf]

PLOS ONE

Frailty does not cause all frail symptoms: United States Health and Retirement Study

--Manuscript Draft--

**Manuscript Number:**	PONE-D-21-33729
**Article Type:**	Research Article
**Full Title:**	Frailty does not cause all frail symptoms: United States Health and Retirement Study
**Short Title:**	Frailty does not cause all frail symptoms
**Corresponding Author:**	Yi-Sheng Chao, M.D., M.P.H., Ph.D.<br>Independent researcher<br>CANADA
**Keywords:**	Frailty; Health and Retirement Study; symptom; correlation; odds ratios
**Abstract:**	Background<br><br>Frailty is associated with major health outcomes. However, the relationships between frailty and frailty symptoms haven't been well studied. This study aims to show the associations between frailty and frailty symptoms.Methods<br><br>The Health and Retirement Study (HRS) is an ongoing longitudinal biannual survey in the United States. Three of the most commonly used frailty diagnoses, defined by the Functional Domains Model, the Burden Model, and the Biologic Syndrome Model, were reproduced according to previous studies. The associations between frailty statuses and input symptoms were assessed using odds ratios and correlation coefficients.Results<br><br>The sample sizes, mean ages, and frailty prevalence matched those reported in previous studies. Frailty statuses were weakly correlated with each other (coefficients = 0.19 to 0.38, $p < 0.001$ for all). There were 49 input symptoms identified by these three models. Frailty statuses defined by the three models were not significantly correlated with one or two symptoms defined by the same models ($p > 0.05$ for all). One to six symptoms defined by the other two models were not significantly correlated with each of the three frailty statuses ($p > 0.05$ for all). Frailty statuses were significantly correlated with their own bias variables ($p < 0.05$ for all).Conclusion<br><br>Frailty diagnoses lack significant correlations with some of their own frailty symptoms and some of the frailty symptoms defined by the other two models. This finding raises questions like whether the frailty symptoms lacking significant correlations with frailty statuses could be included to diagnose frailty and whether frailty exists and causes frailty symptoms.
**Order of Authors:**	Yi-Sheng Chao, M.D., M.P.H., Ph.D.
	Chao-Jung Wu, MSc
	June Y. T. Po, PhD
	Shih-Yu Huang, MD
	Hsing-Chie Wu, MD
	Hui-Ting Hsu, MD
	Yen-Po Cheng, MD
	Yi-Chun Lai, MD
	Wei-Chih Chen, MD
**Additional Information:**	
**Question**	**Response**
**Financial Disclosure**	The authors received no specific funding for this work.

Powered by Editorial Manager® and ProduXion Manager® from Aries Systems Corporation

Enter a financial disclosure statement that describes the sources of funding for the work included in this submission. Review the submission guidelines for detailed requirements. View published research articles from *PLOS ONE* for specific examples.<br><br>This statement is required for submission and **will appear in the published article** if the submission is accepted. Please make sure it is accurate.<br><br>**Unfunded studies**<br>Enter: *The author(s) received no specific funding for this work.*<br><br>**Funded studies**<br>Enter a statement with the following details:<br>• Initials of the authors who received each award<br>• Grant numbers awarded to each author<br>• The full name of each funder<br>• URL of each funder website<br>• Did the sponsors or funders play any role in the study design, data collection and analysis, decision to publish, or preparation of the manuscript?<br>• **NO** - Include this sentence at the end of your statement: *The funders had no role in study design, data collection and analysis, decision to publish, or preparation of the manuscript.*<br>• **YES** - Specify the role(s) played.<br><br>* typeset	
**Competing Interests**<br><br>Use the instructions below to enter a competing interest statement for this submission. On behalf of all authors, disclose any competing interests that could be perceived to bias this work—acknowledging all financial support and any other relevant financial or non-financial competing interests.<br><br>This statement is required for submission and **will appear in the published article** if	YSC is employed by the Canadian Agency for Drugs and Technologies in Health. YSC conducted this study as an independent researcher out of academic curiosity without any material support. No external funding was received for this study. This study is not associated with any patents, products in development or marketed products.

the submission is accepted. Please make sure it is accurate and that any funding sources listed in your Funding Information later in the submission form are also declared in your Financial Disclosure statement.<br><br>View published research articles from *PLOS ONE* for specific examples.<br><br>**NO authors have competing interests**<br><br>Enter: *The authors have declared that no competing interests exist.*<br><br>**Authors with competing interests**<br><br>Enter competing interest details beginning with this statement:<br><br>*I have read the journal's policy and the authors of this manuscript have the following competing interests: [insert competing interests here]*<br><br>* typeset	
**Ethics Statement**<br><br>Enter an ethics statement for this submission. This statement is required if the study involved:<br><br>• Human participants<br>• Human specimens or tissue<br>• Vertebrate animals or cephalopods<br>• Vertebrate embryos or tissues<br>• Field research<br><br>Write "N/A" if the submission does not require an ethics statement.<br><br>General guidance is provided below. Consult the submission guidelines for detailed instructions. **Make sure that all information entered here is included in the Methods section of the manuscript.**	This secondary data analysis was approved by the ethics review committee at the Centre Hospitalier de l'Université de Montréal.

Powered by Editorial Manager® and ProduXion Manager® from Aries Systems Corporation

**Format for specific study types**<br><br>**Human Subject Research (involving human participants and/or tissue)**<br>• Give the name of the institutional review board or ethics committee that approved the study<br>• Include the approval number and/or a statement indicating approval of this research<br>• Indicate the form of consent obtained (written/oral) or the reason that consent was not obtained (e.g. the data were analyzed anonymously)<br><br>**Animal Research (involving vertebrate animals, embryos or tissues)**<br>• Provide the name of the Institutional Animal Care and Use Committee (IACUC) or other relevant ethics board that reviewed the study protocol, and indicate whether they approved this research or granted a formal waiver of ethical approval<br>• Include an approval number if one was obtained<br>• If the study involved *non-human primates*, add *additional details* about animal welfare and steps taken to ameliorate suffering<br>• If anesthesia, euthanasia, or any kind of animal sacrifice is part of the study, include briefly which substances and/or methods were applied<br><br>**Field Research**<br><br>Include the following details if this study involves the collection of plant, animal, or other materials from a natural setting:<br>• Field permit number<br>• Name of the institution or relevant body that granted permission	
**Data Availability**<br><br>Authors are required to make all data underlying the findings described fully available, without restriction, and from the time of publication. PLOS allows rare exceptions to address legal and ethical concerns. See the PLOS Data Policy and FAQ for detailed information.	Yes - all data are fully available without restriction

A Data Availability Statement describing where the data can be found is required at submission. Your answers to this question constitute the Data Availability Statement and **will be published in the article**, if accepted.<br><br>**Important:** Stating 'data available on request from the author' is not sufficient. If your data are only available upon request, select 'No' for the first question and explain your exceptional situation in the text box.<br><br>Do the authors confirm that all data underlying the findings described in their manuscript are fully available without restriction?	
**Describe where the data may be found in full sentences. If you are copying our sample text, replace any instances of XXX with the appropriate details.**<br><br>• If the data are **held or will be held in a public repository**, include URLs, accession numbers or DOIs. If this information will only be available after acceptance, indicate this by ticking the box below. For example: *All XXX files are available from the XXX database (accession number(s) XXX, XXX.*).<br>• If the data are all contained **within the manuscript and/or Supporting Information files**, enter the following: *All relevant data are within the manuscript and its Supporting Information files.*<br>• If neither of these applies but you are able to provide **details of access elsewhere**, with or without limitations, please do so. For example:<br><br>*Data cannot be shared publicly because of [XXX]. Data are available from the XXX Institutional Data Access / Ethics Committee (contact via XXX) for researchers who meet the criteria for access to confidential data.*<br><br>*The data underlying the results presented in the study are available from (include the name of the third party*	The HRS data produced by the RAND Center for the Study of Aging can be accessed via the University of Michigan site (https://hrs.isr.umich.edu/data-products). The authors do not have special access to the HRS data.

Powered by Editorial Manager® and ProduXion Manager® from Aries Systems Corporation

and contact information or URL).<br>• This text is appropriate if the data are owned by a third party and authors do not have permission to share the data.<br><br>* typeset	
Additional data availability information:	

Powered by Editorial Manager® and ProduXion Manager® from Aries Systems Corporation

Click here to access/download;Manuscript;2021_10_18 frail patients are less likely.docx

Frailty does not cause all frail symptoms: United States Health and Retirement Study

Authors

1. Yi-Sheng Chao, MD MPH PhD*
Independent researcher
Email: chaoyisheng@post.harvard.edu
*Corresponding author

2. Chao-Jung Wu, MSc
Université du Québec à Montréal
Email: chao-jung.wu@mail.mcgill.ca

3. June Y. T. Po, PhD
Natural Resources Institute
University of Greenwich
Email: J.Y.T.Po@greenwich.ac.uk

4. Shih-Yu Huang, MD
Department of Anesthesiology
Shuang Ho Hospital
Taipei Medical University
New Taipei City
Department of Anesthesiology
School of Medicine
College of Medicine
Taipei Medical University
Taipei
Email: strik.huang@gmail.com

5. Hsing-Chien Wu, MD
Taipei Hospital
Ministry of Health and Welfare
New Taipei City, Taiwan
Email: s881023@gmail.com

6. Hui-Ting Hsu, MD
Changhua Christian Hospital
Changhua, Taiwan
Email: javawomanfanny@gmail.com

7. Yen-Po Cheng, MD
Changhua Christian Hospital
Email: 134309@cch.org.tw

8. Yi-Chun Lai, MD
National Yang-Ming University Hospital
Email: toto881049@yahoo.com.tw

9. Wei-Chih Chen, MD
Attending Physician
Department of Chest Medicine
Taipei Veterans General Hospital
Lecturer
Institute of Emergency and Critical Care Medicine
National Yang-Ming University
Taipei City, Taiwan
Email: wiji.chen@gmail.com
*Corresponding author

Abstract

Background

Frailty is associated with major health outcomes. However, the relationships between frailty and frailty symptoms haven't been well studied. This study aims to show the associations between frailty and frailty symptoms.

Methods

The Health and Retirement Study (HRS) is an ongoing longitudinal biannual survey in the United States. Three of the most commonly used frailty diagnoses, defined by the Functional Domains Model, the Burden Model, and the Biologic Syndrome Model, were reproduced according to previous studies. The associations between frailty statuses and input symptoms were assessed using odds ratios and correlation coefficients.

Results

The sample sizes, mean ages, and frailty prevalence matched those reported in previous studies. Frailty statuses were weakly correlated with each other (coefficients = 0.19 to 0.38, $p < 0.001$ for all). There were 49 input symptoms identified by these three models. Frailty statuses defined by the three models were not significantly correlated with one or two symptoms defined by the same models ($p > 0.05$ for all). One to six symptoms defined by the other two models were not significantly correlated with each of the three frailty statuses ($p > 0.05$ for all). Frailty statuses were significantly correlated with their own bias variables ($p < 0.05$ for all).

Conclusion

Frailty diagnoses lack significant correlations with some of their own frailty symptoms and some of the frailty symptoms defined by the other two models. This finding raises questions like whether the frailty symptoms lacking significant correlations with frailty statuses could be included to diagnose frailty and whether frailty exists and causes frailty symptoms.

Key words

Frailty; Health and Retirement Study; symptom; correlation; odds ratios

Introduction

Frailty is a geriatric syndrome and can be diagnosed with composite criteria that consist of various frailty symptoms.[1-3] Frailty is often characterized by aging-related symptoms, such as declines in physical and cognitive functioning. It has been considered significant for the prediction of major health outcomes, such as falls, surgical outcomes, and mortality.[2, 3] By aggregating information from multiple symptoms, frailty index scores can be assigned to individuals.[2, 3] Frailty status can then be derived by applying theoretical thresholds to generate frailty index scores.[2, 3] Three of the most commonly used frailty indices require 4 to 70 input domains or symptoms for the diagnosis of frailty.[2]

Ideally, pathological changes or underlying health condition can be identified using diagnoses or proxy measures and are expected to cause or lead to significant increases in symptom occurrence. The significant increases in symptom development can be used to establish diagnoses that represent the diseases or underlying conditions. For example, frailty has been recognized as a cause of disability, independent of clinical conditions.[4] Other researchers also found that frailty can lead to symptoms, particularly mental symptoms[5, 6] and fatigue.[7] However, the effects of frailty on the development of frailty symptoms, those used to diagnose frailty, have not been well discussed. Instead, frailty has been described and defined differently.[1] Some studies have shown how frailty is diagnosed seems far from ideal and lacks pathological confirmation.[2] Researchers have confirmed notable differences in the frail patients identified between the three frailty models.[1]

The causal relationships between frailty and frailty symptoms can be confirmed based on previously published criteria.[1] Among all criteria, the strengths of associations between frailty and frailty symptoms are important and can be used to assess the impact of frailty prevention programs on frailty treatment and to understand the mechanisms that cause frailty. For example, it has been suggested that cognitive impairment plays an important role for frailty diagnosis and mortality among frail patients.[3] The confirmation of the causal relationship between frailty and cognitive function has the potential for intervention development. Without extensive reviews of the relationships between frailty and its symptoms, how frailty may influence frailty symptoms is an important question that is unanswered. This study aims to assess the effect of frailty on the occurrence of frailty symptoms using a cohort that have been used to compare three of the most commonly used frailty indices.

Methods

The Health and Retirement Study (HRS) follows Americans aged 50 years and over every 2 years.[1, 2, 8, 9] The 2004 wave HRS data were used to compare frailty indices defined by 3 models: the Functional Domains Model by Strawbridge et al.,[10] the Burden Model by Rockwood et al.,[11, 12] and the Biologic Syndrome Model by Fried et al.[1, 2, 4] Frailty symptoms or input variables were used to defined various domains.[2-4] When individuals presented enough numbers of frailty symptoms in consideration of input variables in a domain, these individuals might be considered to have a deficit in this domain for the Functional Domain Model and the Biologic Syndrome Model.[2] For example, the weight loss domain in the Biologic Syndrome Model asked individuals whether had body mass index (BMI) less than 18.5 kg/m^2 or whether they lost weight for more than or equal to 10%, compared to two years agoe.[5] This domain required information on weights, heights, BMI, and weights two years ago.[5] In the Burden Model, one symptom represented a single

domain and the presence of a symptom suggested the occurrence of a deficit.[2,5] The frailty indices were the numbers of deficits identified using 4, 70, and 5 domains defined by the 3 frailty models, respectively.[1] Frailty statuses could be diagnosed when individuals had 2, 18 (70 times 0.25), or 3 deficits according to the 3 models, respectively.[1] The details of the frailty symptoms and input variables are published elsewhere (https://doi.org/10.1371/journal.pone.0197859.s002).[2] The names and definitions of the input variables, frailty symptoms, and domains are listed in Table 1 to 3. There were 10, 26, and 14 variables (frailty symptoms, input variables or domains) identified for the 3 models, respectively. In total, there were 57 variables required to produce the frailty indices defined by the 3 models. In addition, there were 4 bias variables induced by the 4 domains in the Functional Domains Model (Table 1), 1 bias variable induced by the Burden Model (Table 2), and 4 bias variables induced by the Biologic Syndrome Model (Table 3).[2]

Statistical analyses

The associations between the frailty statuses and itheir nput symptoms were determined with odds ratios and correlation coefficients. Odds ratios were the ratios of the odds that an outcome of developing symptoms occurred among frail individuals, compared to the odds among those not frail.[13] Odds ratios were applicable to binomial variables.[13] Odds ratios equaling 1 suggest that the two groups have similar risks of developing symptoms.[13] The processing to transform non-binomial variables to binomial variables were according to the authors of the Burden Model.[14] Pearson's correlation coefficients were used to assess the associations between frailty statuses defined by the 3 models and frailty symptoms or input variables or domains or bias variables.[15] Correlation coefficients ranged from -1 to 1, representing completely opposite information and identical information between 2 variables, respectively. We hypothesized that I) frailty statuses were not associated with symptom incidence (odds ratio = 1); ii) frailty statuses were not correlated with frailty symptoms or input variables of the frailty indices (correlation coefficient = 0). Correlation coefficients between 0 and 0.10, 0.10 and 0.39, 0.40 and 0.69, 0.70 and 0.89, and 0.90 and 1.00 were interpreted as negligible, weak, moderate, strong, and very strong correlations, respectively.[16] P values were adjusted for multiple comparison using false discovery rates.[17] Two-tailed P values that were less than 0.05 were considered statistically significant. All statistical analyses were conducted within R environment (v4.0.4)[18] and RStudio (v1.4.1106).[19]

Results

There were 11,113, 7,713, and 1,642 HRS participants analyzed for the frailty indices defined by the Functional Domains Model, the Burden Model, and the Biologic Syndrome Model in Table 1 to 3, respectively.[2] The numbers of frail patients were 3,059 (27.53%), 3,442 (44.63%), and 203 (12.36%), respectively.[2] The mean ages were 74.92, 78.43, and 77.05 years, respectively. The proportions of females were 57.46%, 58.78%, and 54.69%, respectively.

Frailty symptom development based on frailty status

In Table 1 to 3, the associations between frailty status (yes or no) and symptom development are shown using odds ratios and correlation coefficients. Overall, most of frailty symptoms were significantly associated with frailty statuses. However, frailty statuses defined by the three models were not significantly associated with all frailty symptoms or input variables or domains. The frailty symptoms or input variables or domains that were not significantly associated with frailty statuses are described below. The correlation

coefficients between the three frailty statuses ranged from 0.19 to 0.38 (weak correlations, $p < 0.001$ for all).

In Table 1, the frailty status defined by the Functional Domains Model was not significantly correlated with one input variable, BMI (correlation coefficients = -0.02, 95% CI = -0.04 to 0).

Among the frailty symptoms or input variables or domains identified by the other two models, two symptoms, malignant disease and tiredness all the time, were not significantly associated with this frailty status (p of correlations > 0.05 for both symptoms). Among the bias variables, one bias variable that was induced by having one of two Center for Epidemiologic Studies Depression (CES-D) items was not significantly associated with this frailty status (correlation $p > 0.05$).

In Table 2, the frailty status defined by the Burden Model was assessed for the associations with frailty symptoms, input variables, and domains. One input symptom, tiredness all the time, was not significantly associated with this frailty status (p of correlation > 0.05). One symptom identified by the other two models, self-reported weight, was not significantly correlated with this frailty status ($p > 0.05$). Among the bias variables, four were not significantly correlated with this frailty status ($p > 0.05$ for all).

In Table 3, the frailty status defined by the Biologic Syndrome Model was assessed for the associations with frailty symptoms, input variables, and domains. BMI was not significantly correlated with this frailty status ($p > 0.05$). Because the values of two symptoms, proxy memory rating and history of stroke, were the same for frail and non-frail HRS participants for this frailty index, their correlations with this frailty status could not be assessed. Six symptoms defined by the other two models, history of malignant disease, diabetes mellitus, headache, memory change, musculoskeletal problems, and change in general mental functioning, were not significantly correlated with this frailty status ($p > 0.05$ for all).

Correlations with bias variables

In Table 1 to 3, the correlations with bias variables are shown for the three frailty indices. Each frailty status was significantly associated with the bias variables induced by their own diagnostic criteria. The frailty statuses defined by the Functional Domains Model, the Burden Model, and the Biologic Syndrome Model, were significantly correlated with four, one, and four bias variables induced by their own models, respectively ($p < 0.05$ for all). In addition, the frailty status defined by the Functional Domains Model, the Burden Model, and the Biologic Syndrome Model, were significantly correlated with three, four, and two bias variables induced by the other two models, respectively ($p < 0.05$ for all).

Discussion

Strengths of the associations are one of the Bradford-Hill criteria to assess whether a disease causes symptoms or outcomes.[20] Frailty has been promising in establishing causal relationships with major health outcomes, such as mortality and falls, based on frailty's significant associations with them.[2] However, whether frailty causes frailty symptoms have not been well studied. In this study using the HRS data, three of the most commonly used frailty diagnoses fail to demonstrate significant correlations with some of the frailty symptoms of their own or those defined by the other two frailty diagnoses. When frailty lacks significant associations with frailty symptoms, this suggests frailty diagnoses are made based on so-called frailty symptoms, some of which frailty may not cause them. This needs serious discussion and examination.

The pathological changes that are considered related to frailty include sarcopenia, heart disease, and lung disease, depending on the frailty models.[2] In this study, frailty diagnoses do not fully support the models of their own by showing insignificant correlations with some of their frailty symptoms or input variables. This issue becomes more problematic for frailty diagnoses made based on a large number of input symptoms. The frailty status defined by the Burden Model requires at least 30 symptoms for diagnosis and 70 symptoms have been often used.[14] This frailty status is not significantly associated with two of its input symptoms in this study. The role of insignificant symptoms, seizure and tiredness, in frailty status defined by the Burden Model haven't been discussed in previous studies.[14]

The differences in the assumptions between frailty models can be shown with the symptoms that best explained frailty statuses. We found the frailty symptoms or input variables that had the largest correlation coefficients with the three frailty statuses were different. The three symptoms that have the largest significant correlation coefficients with the frailty indices defined by the Functional Domain Model, the Burden Model, and the Biologic Syndrome Model are some difficulties in lifting 10 pounds, some difficulty in mobility, and slowness measured by time to walk 8 feet are the symptoms, respectively. The paths from non-frailty to frailty vary depending on the models used.

Moreover, the three frailty diagnoses are subject to bias variables induced by inadequate data processing.[2] These biases can occur due to censoring the sum of multiple input variables, inadequate data transformation, or categorization of continuous data.[2, 21] The bias variables induced by the Biologic Syndrome Model have been shown to better explain the frailty diagnosis than its frailty symptoms.[2] Interestingly, the bias variables are correlated not only with their own frailty diagnoses, but also the frailty diagnoses defined by the other two models. How these bias variables affect the correlations between frailty diagnoses and their input symptoms remains a question for further research.

In sum, the results highlight logic challenges. Frail patients are not more likely to have certain frail symptoms, but these symptoms are necessary to make these diagnoses. It is unclear whether the symptoms that frailty is insignificantly correlated with can be called "frailty" symptom or used for frailty diagnosis. When excluding these symptoms from being used for the diagnosis of frailty, the prevalence of frailty decreases. Many of the published frailty prevalence rates are likely to be overestimated, because we have not identified any studies explicitly examine the significance of the associations between the frailty statuses and frailty symptoms they defined in their own models. We will continue exploring the causal relationship between frailty and frailty symptoms using other data sets in the future.

Limitations

This study has strengths in using a publicly accessible database that has been investigated in previous studies.[1, 2] The demographic characteristics reported in this study matched those reported.[1] However, there are several limitations to this study. There are other statistical and epidemiological measures of association that can be tested to demonstrate the strengths of associations, including Chi-squared statistics and risk ratios.[22, 23] Odds ratios are adequate for cross-sectional studies to approximate risk ratios.[13] However, odds ratios can over- or under-estimate effect sizes if the underlying risk ratios are greater or less than 1, respectively.[24] Other measures of association will be explored in the future. Moreover, there are other factors influencing the correlations between frailty statuses and frailty symptoms, including demographic characteristics. These

factors can be adjusted using techniques, such as multiple regression.[25, 26] This will need to be explored in future research.

Conclusion

Three of the frailty diagnoses defined by three models were assessed for their correlations with frailty symptoms of their own, those defined by the other two models, and bias variables using odds ratios and correlation coefficients. Frailty diagnoses lack significant correlations with some of their own frailty symptoms and some of the frailty symptoms defined by the other two models. This suggests that frail patients are not more likely to have certain frailty symptoms using any of the three frailty models. This finding raises questions like whether frailty symptoms lacking significant correlations with frailty statuses could be included to diagnose frailty and whether frailty exists and causes frailty symptoms. Further research to assess the causal relationships between frailty and frailty symptoms is needed and planned.

Declaration

Consent for publication

The consent for publication was not required for this secondary data analysis.

Acknowledgements

Not applicable.

Competing interests

YSC is employed by the Canadian Agency for Drugs and Technologies in Health. YSC conducted this study as an independent researcher out of academic curiosity without any material support. No external funding was received for this study. This study is not associated with any patents, products in development or marketed products

Authors' contribution

YSC conceptualized and designed this study, managed and analyzed data, and drafted the manuscript. CJW assisted in data management and computation. JYTP, SYH, HCW, HTH, YPC, YCL, and WCC participated in the design of this study. All authors reviewed and approved the manuscript.

Availability of data and material

The HRS data produced by the RAND Center for the Study of Aging can be accessed via the University of Michigan site (https://hrs.isr.umich.edu/data-products). The authors do not have special access to the HRS data.

Funding

No specific funding for this study.

Ethics review

This secondary data analysis was approved by the ethics review committee at the Centre Hospitalier de l'Université de Montréal.

References

1. Cigolle CT, Ofstedal MB, Tian Z, Blaum CS. Comparing models of frailty: the Health and Retirement Study. J Am Geriatr Soc. 2009;57(5):830-9. Epub 2009/05/21. doi: 10.1111/j.1532-5415.2009.02225.x.

2. Chao Y-S, Wu H-C, Wu C-J, Chen W-C. Index or illusion: The case of frailty indices in the Health and Retirement Study. PLOS ONE. 2018;13(7):e0197859. doi: 10.1371/journal.pone.0197859.

3. Chao Y-S, Wu C-J, Wu H-C, Hsu H-T, Tsao L-C, Cheng Y-P, et al. Composite diagnostic criteria are problematic for linking potentially distinct populations: the case of frailty. Scientific Reports. 2020;10(1):2601. doi: 10.1038/s41598-020-58782-1.

4. Fried LP, Tangen CM, Walston J, Newman AB, Hirsch C, Gottdiener J, et al. Frailty in Older Adults: Evidence for a Phenotype. The Journals of Gerontology Series A: Biological Sciences and Medical Sciences. 2001;56(3):M146-M57. doi: 10.1093/gerona/56.3.M146.

5. St. John PD, Tyas SL, Montgomery PR. Depressive symptoms and frailty. International journal of geriatric psychiatry. 2013;28(6):607-14.

6. Nascimento PPP, Batistoni SST, Neri AL. Frailty and depressive symptoms in older adults: data from the FIBRA study-UNICAMP. Psicologia: Reflexão e Crítica. 2016;29.

7. Uslu A, Canbolat O, editors. Relationship Between Frailty and Fatigue in Older Cancer Patients2021: Elsevier.

8. RAND Corporation. RAND HRS Data Files, supported by NIA and SSA Santa Monica, CA: RAND Corporation,; 2016 [updated September 2016; cited 2016 Nov 29]. Available from: http://www.rand.org/labor/aging/dataprod/hrs-data.html.

9. Health and Retirement Study, RAND HRS Data File (v.P) public use dataset. In: U01AG009740). PadbtUoMwfftNIoAgnN, editor. Ann Arbor, MI2016.

10. Strawbridge WJ, Shema SJ, Balfour JL, Higby HR, Kaplan GA. Antecedents of frailty over three decades in an older cohort. J Gerontol B Psychol Sci Soc Sci. 1998;53(1):S9-16. Epub 1998/02/20.

11. Rockwood K, Andrew M, Mitnitski A. A comparison of two approaches to measuring frailty in elderly people. J Gerontol A Biol Sci Med Sci. 2007;62(7):738-43. Epub 2007/07/20.

12. Rockwood K, Mitnitski A. Frailty in relation to the accumulation of deficits. J Gerontol Biol Sci Med Sci. 2007;62A. doi: 10.1093/gerona/62.7.722.

13. Rothman KJ, Greenland S, Lash TL. Modern Epidemiology. New York, NY: Wolters Kluwer Health/Lippincott Williams & Wilkins; 2008.

14. Searle SD, Mitnitski A, Gahbauer EA, Gill TM, Rockwood K. A standard procedure for creating a frailty index. BMC Geriatrics. 2008;8(1):24. doi: 10.1186/1471-2318-8-24.

15. Belhekar VM. Statistics for Psychology Using R: SAGE Publications; 2016.

16. Schober P, Boer C, Schwarte LA. Correlation coefficients: appropriate use and interpretation. Anesthesia & Analgesia. 2018;126(5):1763-8.

17. Benjamini Y, Hochberg Y. Controlling the false discovery rate: a practical and powerful approach to multiple testing. Journal of the Royal statistical society: series B (Methodological). 1995;57(1):289-300.

18. R Development Core Team. R: A language and environment for statistical computing. Vienna, Austria: R Foundation for Statistical Computing; 2016.

19. RStudio Team. RStudio: Integrated Development for R. Boston, MA: RStudio, Inc.; 2016.

20. Hill AB. The environment and disease: association or causation? : Sage Publications; 1965.

21. Chao Y-S, Lin K-F, Wu C-J, Wu H-C, Hsu H-T, Tsao L-C, et al. Simulation study to demonstrate biases created by diagnostic criteria of mental illnesses: major depressive episodes, dysthymia, and manic episodes. BMJ Open. 2020;10(11):e037022. doi: 10.1136/bmjopen-2020-037022.
22. Argyrous G. Introduction to measures of association. Statistics for Social Research: Springer; 1997. p. 313-8.
23. Khamis H. Measures of association: how to choose? Journal of Diagnostic Medical Sonography. 2008;24(3):155-62.
24. Zhang J, Yu KF. What's the Relative Risk?A Method of Correcting the Odds Ratio in Cohort Studies of Common Outcomes. JAMA. 1998;280(19):1690-1. doi: 10.1001/jama.280.19.1690.
25. Hastie T, Tibshirani R, Friedman J. The Elements of Statistical Learning: Data Mining, Inference, and Prediction, Second Edition: Springer New York; 2009.
26. James G, Witten D, Hastie T, Tibshirani R. An Introduction to Statistical Learning: with Applications in R: Springer New York; 2013.

Table 1. Frailty status defined by the Functional Domains Model and its associations with frailty symptoms

HRS variables	Definitions	N with symptoms for binomial variables	Odds ratios (95% CIs)	Mean (SD)	Correlation coefficients (95% CIs)
**r7agey_b**	Age at interview (years)			73.92 (7.59)	0.26 (0.24 to 0.27)***
**r7bmi**	Self-reported body mass index=kg/m2			26.68 (5.31)	-0.02 (-0.04 to 0)
**r7cogtot**	Total cognition summary score			20.53 (6.08)	-0.41 (-0.42 to -0.39)***
**r7dizz**	Physical functioning: Dizziness as persistent problem	1577	7.9 (7.03 to 8.87)***	0.14 (0.35)	0.36 (0.35 to 0.38)***
**r7eye**	Sensory problems: Fair or poor eyesight despite use of corrective lenses			2.95 (1.01)	0.44 (0.42 to 0.45)***
**r7fall**	fallen down last 2 years			0.9 (2.88)	0.29 (0.27 to 0.31)***
**r7frailim1**	Frailty index: Functional Domains Model			0.98 (0.93)	0.85 (0.84 to 0.85)***
**r7frailim1cat**	Frailty status: Functional Domains Model (outcome of this table)	3059	Not applicable	0.28 (0.45)	1 (1 to 1)***
**r7hear**	Sensory problems: fair or poor hearing despite use of hearing aides			2.87 (1.12)	0.37 (0.35 to 0.39)***
**r7lift**	some difficulty in lift/carry 10lbs	3631	8.85 (8.05 to 9.72)***	0.33 (0.47)	0.46 (0.45 to 0.48)***
**r7memopr**	Proxy memory rating			1.39 (1.18)	0.33 (0.31 to 0.35)***
**r7wchange**	Weight in wave 2002 minus weight in wave 2004 (%)			0.01 (0.08)	-0.04 (-0.05 to -0.02)***
**ragender**	Male = 0; female = 1	6385	1.32 (1.21 to 1.44)***	0.57 (0.49)	0.06 (0.04 to 0.08)***
**Domains and other frailty symptoms identified by the other 2 models**					
**r7actsum**	Summary scores of physical activities			33.45 (9.11)	0.31 (0.29 to 0.33)***
**r7arthrcat**	Binomial: Arthritis	7693	1.84 (1.67 to 2.03)***	0.69 (0.46)	0.12 (0.1 to 0.14)***
**r7bathcat**	Binomial: Problems with bathing	1264	8.28 (7.27 to 9.42)***	0.11 (0.32)	0.34 (0.32 to 0.36)***
**r7cancrcat**	Binomial: Malignant disease	1978	1.03 (0.92 to 1.15)	0.18 (0.38)	0.01 (-0.01 to 0.02)
**r7cogimpair**	Impaired cognition based on performance-based scores or proxy assessment	999	23.09 (19.14 to 27.85)***	0.09 (0.29)	0.41 (0.4 to 0.43)***
**r7deprescat**	Binomial: Feeling sad, blue, depressed	2011	3.54 (3.2 to 3.91)***	0.18 (0.39)	0.24 (0.22 to 0.26)***
**r7diabscat**	Binomial: History of diabetes mellitus	292	1.38 (1.08 to 1.77)*	0.03 (0.16)	0.02 (0.01 to 0.04)**
**r7dresscat**	Binomial: problem getting dressed	1419	6.01 (5.34 to 6.76)***	0.13 (0.33)	0.31 (0.29 to 0.32)***
**r7effort**	everything an effort	2932	3.96 (3.62 to 4.34)***	0.26 (0.44)	0.29 (0.27 to 0.31)***
**r7fall_cat1**	More than 1 falls	3640	3.6 (3.3 to 3.93)***	0.33 (0.47)	0.28 (0.26 to 0.3)***
**r7fall_cat2**	More than 2 falls	1929	6.71 (6.04 to 7.46)***	0.17 (0.38)	0.36 (0.35 to 0.38)***
**r7frail1_1**	Dizziness as persistent problem, >=2 falls in previous 2 years, or difficulty lifting 10 pounds	4383	27.81 (24.63 to 31.42)***	0.39 (0.49)	0.61 (0.6 to 0.62)***
**r7frail1_2**	Weight in wave 2002 minus weight in wave 2004 !10% of weight in wave 2002 or body mass index o18.5 kg/m2	866	9.35 (7.97 to 10.96)***	0.08 (0.27)	0.3 (0.29 to 0.32)***
**r7frail1_3**	Mild to severe cognitive impairment on performance-based measure or according to proxy and interviewer rating			0.09 (0.29)	0.42 (0.4 to 0.43)***
**r7frail1_4**	Fair or poor eyesight despite use of corrective lenses or fair or poor hearing despite use of hearing aides			0.42 (0.49)	0.59 (0.58 to 0.6)***
**r7frail3_2**	Yes to either of two CES-D items: (i) Felt that everything I did was an effort in last week. (ii) Could not get going in last week.	4025	3.99 (3.66 to 4.35)***	0.36 (0.48)	0.3 (0.29 to 0.32)***
**r7frail3_3**	Frequency of three intensities of activity, lowest quintile (stratified according to sex)	2872	4.11 (3.75 to 4.51)***	0.26 (0.44)	0.3 (0.28 to 0.32)***
**r7frail3_4**	Time to walk 8 ft, converted to time to walk 15 ft. Cutoff criteria according to sex and height remain the same	5474	1.44 (1.33 to 1.57)***	0.49 (0.5)	0.08 (0.06 to 0.1)***
**r7frail3_5**	Grip strength: Weakest 20% (stratified according to sex and BMI)	2529	1.43 (1.3 to 1.57)***	0.23 (0.42)	0.07 (0.05 to 0.09)***
**r7frailim1**	Frailty index: Functional Domains Model			0.98 (0.93)	0.85 (0.84 to 0.85)***
**r7frailim2**	Frailty index: Burden Model			5.02 (2.83)	0.46 (0.44 to 0.47)***
**r7frailim2cat**	Frailty status: Burden Model			0.45 (0.5)	0.38 (0.36 to 0.4)***

**r7frailim3**	Frailty index: Biologic Syndrome Model			1.14 (1.08)	0.35 (0.3 to 0.39)***
**r7frailim3cat**	Frailty status: Biologic Syndrome Model			0.12 (0.33)	0.3 (0.26 to 0.34)***
**r7going**	could not get going	2682	3.41 (3.11 to 3.73)***	0.24 (0.43)	0.25 (0.24 to 0.27)***
**r7grip**	Grip strength, largest value			30.19 (16.32)	-0.07 (-0.09 to -0.06)***
**r7gripl**	Grip strength, left hand			26.63 (16.29)	-0.07 (-0.09 to -0.05)***
**r7gripr**	Grip strength, right hand			28.74 (16.14)	-0.1 (-0.12 to -0.08)***
**r7headac**	Headache	817	3.05 (2.64 to 3.53)***	0.07 (0.26)	0.15 (0.13 to 0.17)***
**r7heartcat**	Binomial: Cardiac problems	3631	2.15 (1.97 to 2.35)***	0.33 (0.47)	0.17 (0.15 to 0.18)***
**r7height**	Self-reported height in meters			1.68 (0.1)	-0.09 (-0.11 to -0.07)***
**r7hibp**	had high blood pressure since last interview	6791	1.49 (1.36 to 1.62)***	0.61 (0.49)	0.08 (0.07 to 0.1)***
**r7ltactx**	Frequency of light physical activity			2.87 (1.2)	0.29 (0.28 to 0.31)***
**r7lungcat**	Binomial: Lung problems	1345	2.02 (1.79 to 2.27)***	0.12 (0.33)	0.11 (0.09 to 0.13)***
**r7mdactx**	Frequency of moderate physical activity			3.13 (1.37)	0.29 (0.27 to 0.31)***
**r7memrysca t**	Binomial: Memory changes	345	7.46 (5.86 to 9.48)***	0.03 (0.17)	0.18 (0.16 to 0.2)***
**r7mobila**	Some difficulty in mobility /05			1.36 (1.59)	0.43 (0.42 to 0.45)***
**r7muscle**	Musculoskeletal problems	386	1.3 (1.05 to 1.62)*	0.03 (0.18)	0.02 (0 to 0.04)*
**r7psychcat**	Binomial: Depression	1799	2.82 (2.55 to 3.13)***	0.16 (0.37)	0.19 (0.17 to 0.21)***
**r7psychscat**	Binomial: Changes in general mental functioning	246	3.6 (2.78 to 4.64)***	0.02 (0.15)	0.1 (0.08 to 0.12)***
**r7seizure**	Seizures, generalized			()	
**r7sleeprcat**	Binomial: Sleep changes	3102	2.26 (2.07 to 2.47)***	0.28 (0.45)	0.17 (0.15 to 0.19)***
**r7strokcat**	Binomial: Cerebrovascular problems	1139	3.36 (2.97 to 3.81)***	0.1 (0.3)	0.19 (0.17 to 0.21)***
**r7strokecat**	Binomial: History of stroke	1283	3.39 (3.01 to 3.82)***	0.12 (0.32)	0.2 (0.18 to 0.22)***
**r7stroks**	had stroke since last interview	255	3.31 (2.58 to 4.25)***	0.02 (0.15)	0.09 (0.08 to 0.11)***
**r7tired**	Tiredness all the time	1	Not applicable	0 (0.01)	-0.01 (-0.02 to 0.01)
**r7toiltcat**	Binomial: Toileting problems	903	6.9 (5.95 to 8)***	0.08 (0.27)	0.27 (0.26 to 0.29)***
**r7underw**	Underweight in wave 2004 (%)	313	7.94 (6.15 to 10.25)***	0.03 (0.17)	0.18 (0.16 to 0.19)***
**r7urine**	Urinary incontinence	2710	2.35 (2.15 to 2.58)***	0.24 (0.43)	0.18 (0.16 to 0.19)***
**r7vgactx**	Frequency of vigorous physical activity			4.24 (1.24)	0.2 (0.18 to 0.22)***
**r7walkt**	Slowness: Time to walk 8 ft, converted to time to walk 15 ft. Cutoff criteria according to sex and height remain the same			5.09 (19.08)	0.11 (0.1 to 0.13)***
**r7walkt15**	Time to walk 15 feet			9.54 (35.77)	0.11 (0.1 to 0.13)***
**r7weight**	Self-reported weight in kilograms			76.03 (17.43)	-0.06 (-0.08 to -0.04)***
**Bias variables**					
**Biases induced by the Functional Domains Model**					
**r7frail1_1res**	Bias induced by Dizziness as persistent problem, >=2 falls in previous 2 years, or difficulty lifting 10 pounds			0 (0.29)	0.35 (0.34 to 0.37)***
**r7frail1_2res**	Bias induced by Weight in wave 2002 minus weight in wave 2004 !10% of weight in wave 2002 or body mass index o18.5 kg/m2			0 (0.25)	0.2 (0.19 to 0.22)***
**r7frail1_3res**	Bias induced by Mild to severe cognitive impairment on performance-based measure or according to proxy and interviewer rating			0 (0.15)	-0.03 (-0.05 to -0.01)**
**r7frail1_4res**	Bias induced by Fair or poor eyesight despite use of corrective lenses or fair or poor hearing despite use of hearing aides			0 (0.2)	0.02 (0.01 to 0.04)*
**Biases induced by the Burden Model**					
**r7frail2res**	Bias induced by Summary of proxy memory rating and total cognition summary score			0 (0.04)	-0.14 (-0.16 to -0.11)***
**Biases induced by the Biologic Syndrome Model**					

**r7frail3_2res**	Bias induced by Yes to either of two CES-D items: (i) Felt that everything I did was an effort in last week. (ii) Could not get going in last week.			0 (0.17)	0.01 (-0.04 to 0.06)
**r7frail3_3res**	Bias induced by Frequency of three intensities of activity, lowest quintile (stratified according to sex)			0 (0.31)	0.1 (0.05 to 0.15)***
**r7frail3_4res**	Bias induced by Time to walk 8 ft, converted to time to walk 15 ft. Cutoff criteria according to sex and height remain the same			0 (0.49)	0.16 (0.12 to 0.21)***
**r7frail3_5res**	Bias induced by Grip strength: Weakest 20% (stratified according to sex and BMI)			0 (0.34)	0.05 (0 to 0.09)

n = 11,113; frailty n (%) = 3,059 (27.53%); mean age = 74.92 years; female % = 57.46%.
BMI = body mass index; CES-D = Center for Epidemiological Studies Depression; HRS = Health and Retirement Study.
* = $p < 0.05$; ** = $p < 0.01$; *** = $p < 0.001$.

Table 2. Frailty status defined by the Burden Model and its associations with frailty symptoms

HRS variables	Definitions	N with symptoms for binomial variables	Odds ratios (95% CIs	Mean (SD)	Correlation coefficients (95% CIs)
**r7agey_b**	Age at interview (years)			77.43 (6.46)	0.19 (0.17 to 0.22)***
**r7arthrcat**	Binomial: Arthritis	5457	4.45 (3.97 to 4.99)***	0.71 (0.45)	0.3 (0.28 to 0.32)***
**r7bathcat**	Binomial: Problems with bathing	1091	28.44 (21.95 to 36.86)***	0.14 (0.35)	0.4 (0.39 to 0.42)***
**r7cancrcat**	Binomial: Malignant disease	1489	2.12 (1.89 to 2.38)***	0.19 (0.39)	0.15 (0.13 to 0.17)***
**r7cogtot**	Total cognition summary score			19.69 (6.27)	-0.25 (-0.27 to -0.23)***
**r7deprescat**	Binomial: Feeling sad, blue, depressed	1480	5.55 (4.87 to 6.32)***	0.19 (0.39)	0.31 (0.29 to 0.33)***
**r7diabscat**	Binomial: History of diabetes mellitus	174	3.07 (2.21 to 4.26)***	0.02 (0.15)	0.08 (0.06 to 0.1)***
**r7dresscat**	Binomial: problem getting dressed	1159	18.97 (15.34 to 23.48)***	0.15 (0.36)	0.4 (0.38 to 0.42)***
**r7fall**	fallen down last 2 years			0.99 (2.89)	0.3 (0.28 to 0.32)***
**r7frailim2**	Frailty index: Burden Model			5.02 (2.83)	0.8 (0.8 to 0.81)***
**r7frailim2cat**	Frailty status: Burden Model (outcome of this table)	3442	Not applicable	0.45 (0.5)	1 (1 to 1)***
**r7headac**	Headache	514	5.28 (4.25 to 6.58)***	0.07 (0.25)	0.19 (0.17 to 0.21)***
**r7heartcat**	Binomial: Cardiac problems	2817	4.01 (3.64 to 4.42)***	0.37 (0.48)	0.32 (0.3 to 0.34)***
**r7hibp**	had high blood pressure since last interview	4809	2.81 (2.55 to 3.1)***	0.62 (0.48)	0.24 (0.22 to 0.26)***
**r7lungcat**	Binomial: Lung problems	928	4.41 (3.77 to 5.16)***	0.12 (0.33)	0.23 (0.2 to 0.25)***
**r7memopr**	Proxy memory rating			1.45 (1.29)	0.23 (0.21 to 0.25)***
**r7memryscat**	Binomial: Memory changes	303	5.95 (4.43 to 8)***	0.04 (0.19)	0.15 (0.13 to 0.17)***
**r7mobila**	Some difficulty in mobility /05			1.51 (1.66)	0.56 (0.55 to 0.58)***
**r7muscle**	Musculoskeletal problems	258	3.61 (2.73 to 4.78)***	0.03 (0.18)	0.11 (0.09 to 0.13)***
**r7psychcat**	Binomial: Depression	1230	7.74 (6.63 to 9.04)***	0.16 (0.37)	0.33 (0.31 to 0.35)***
**r7psychscat**	Binomial: Changes in general mental functioning	188	15.02 (8.84 to 25.5)***	0.02 (0.15)	0.15 (0.13 to 0.17)***
**r7seizure**	Seizures, generalized	0	Not applicable	0	Not applicable for uniform values
**r7sleeprcat**	Binomial: Sleep changes	2185	5.05 (4.53 to 5.63)***	0.28 (0.45)	0.35 (0.33 to 0.37)***
**r7strokcat**	Binomial: Cerebrovascular problems	923	11.45 (9.34 to 14.03)***	0.12 (0.32)	0.32 (0.3 to 0.34)***
**r7strokecat**	Binomial: History of stroke	1062	10.42 (8.68 to 12.51)***	0.14 (0.34)	0.34 (0.32 to 0.36)***
**r7tired**	Tiredness all the time	1	Not applicable	0 (0.01)	0.01 (-0.01 to 0.03)
**r7toiltcat**	Binomial: Toileting problems	759	44.18 (29.79 to 65.52)***	0.1 (0.3)	0.35 (0.33 to 0.36)***
**r7urine**	Urinary incontinence	2075	6.41 (5.71 to 7.19)***	0.27 (0.44)	0.38 (0.36 to 0.4)***
**ragender**	Male = 0; female = 1	4534	1.69 (1.54 to 1.85)***	0.59 (0.49)	0.13 (0.1 to 0.15)***
**Domains and other frailty symptoms identified by the other 2 models**					
**r7actsum**	Summary scores of physical activities			34.44 (8.92)	0.35 (0.33 to 0.37)***
**r7bmi**	Self-reported body mass index=kg/m2			26.1 (5.07)	0.07 (0.05 to 0.1)***
**r7cogimpair**	Impaired cognition based on performance-based scores or proxy assessment	884	4.71 (4 to 5.54)***	0.11 (0.32)	0.23 (0.21 to 0.25)***
**r7dizz**	Physical functioning: Dizziness as persistent problem	1163	3.93 (3.42 to 4.5)***	0.15 (0.36)	0.23 (0.21 to 0.25)***
**r7effort**	Everything an effort	2153	4.59 (4.12 to 5.11)***	0.28 (0.45)	0.33 (0.31 to 0.35)***
**r7eye**	Sensory problems: Fair or poor eyesight despite use of corrective lenses			3 (1.03)	0.26 (0.24 to 0.28)***
**r7fall_cat1**	More than 1 falls	2761	5.61 (5.06 to 6.21)***	0.36 (0.48)	0.39 (0.37 to 0.41)***

**r7fall_cat2**	More than 2 falls	1499	6.94 (6.06 to 7.95)***	0.19 (0.4)	0.35 (0.33 to 0.37)***
**r7frail1_1**	Dizziness as persistent problem, >=2 falls in previous 2 years, or difficulty lifting 10 pounds	3290	6.02 (5.45 to 6.64)***	0.43 (0.49)	0.42 (0.4 to 0.44)***
**r7frail1_2**	Weight in wave 2002 minus weight in wave 2004 !10% of weight in wave 2002 or body mass index o18.5 kg/m2	635	1.97 (1.67 to 2.32)***	0.08 (0.27)	0.09 (0.07 to 0.11)***
**r7frail1_3**	Mild to severe cognitive impairment on performance-based measure or according to proxy and interviewer rating			0.11 (0.32)	0.23 (0.21 to 0.25)***
**r7frail1_4**	Fair or poor eyesight despite use of corrective lenses or fair or poor hearing despite use of hearing aides			0.45 (0.5)	0.22 (0.2 to 0.24)***
**r7frail3_2**	Yes to either of two CES-D items: (i) Felt that everything I did was an effort in last week. (ii) Could not get going in last week.	2977	4.72 (4.28 to 5.21)***	0.39 (0.49)	0.36 (0.34 to 0.38)***
**r7frail3_3**	Frequency of three intensities of activity, lowest quintile (stratified according to sex)	2226	4.89 (4.39 to 5.45)***	0.29 (0.45)	0.34 (0.32 to 0.36)***
**r7frail3_4**	Time to walk 8 ft, converted to time to walk 15 ft. Cutoff criteria according to sex and height remain the same	3853	1.37 (1.25 to 1.5)***	0.5 (0.5)	0.08 (0.06 to 0.1)***
**r7frail3_5**	Grip strength: Weakest 20% (stratified according to sex and BMI)	1840	1.4 (1.26 to 1.55)***	0.24 (0.43)	0.07 (0.05 to 0.09)***
**r7frailim1**	Frailty index: Functional Domains Model			1.08 (0.96)	0.43 (0.41 to 0.45)***
**r7frailim1cat**	Frailty status: Functional Domains Model	2434	5.57 (5.01 to 6.19)***	0.32 (0.46)	0.38 (0.36 to 0.4)***
**r7frailim2**	Frailty index: Burden Model			5.02 (2.83)	0.8 (0.8 to 0.81)***
**r7frailim3**	Frailty index: Biologic Syndrome Model			1.22 (1.11)	0.28 (0.23 to 0.33)***
**r7frailim3cat**	Frailty status: Biologic Syndrome Model			0.14 (0.35)	0.19 (0.14 to 0.24)***
**r7going**	could not get going	2026	4.64 (4.15 to 5.18)***	0.26 (0.44)	0.32 (0.3 to 0.34)***
**r7grip**	Grip strength, largest value			29.49 (15.93)	-0.08 (-0.1 to -0.06)***
**r7gripl**	Grip strength, left hand			25.89 (15.72)	-0.07 (-0.09 to -0.04)***
**r7gripr**	Grip strength, right hand			27.99 (15.37)	-0.11 (-0.13 to -0.09)***
**r7hear**	Sensory problems: fair or poor hearing despite use of hearing aides			2.97 (1.13)	0.16 (0.14 to 0.18)***
**r7height**	Self-reported height in meters			1.68 (0.1)	-0.1 (-0.13 to -0.08)***
**r7lift**	Some difficulty in lift/carry 10lbs	2862	5.88 (5.31 to 6.51)***	0.37 (0.48)	0.4 (0.39 to 0.42)***
**r7ltactx**	Frequency of light physical activity			2.99 (1.24)	0.3 (0.28 to 0.32)***
**r7mdactx**	Frequency of moderate physical activity			3.24 (1.41)	0.34 (0.32 to 0.36)***
**r7stroks**	had stroke since last interview	208	13 (8.09 to 20.88)***	0.03 (0.16)	0.15 (0.13 to 0.18)***
**r7underw**	Underweight in wave 2004 (%)	270	1.68 (1.31 to 2.14)***	0.04 (0.18)	0.05 (0.03 to 0.07)***
**r7vgactx**	Frequency of vigorous physical activity			4.35 (1.19)	0.23 (0.21 to 0.25)***
**r7walkt**	Slowness: Time to walk 8 ft, converted to time to walk 15 ft. Cutoff criteria according to sex and height remain the same			5.2 (19.49)	0.12 (0.1 to 0.15)***
**r7walkt15**	Time to walk 15 feet			9.75 (36.55)	0.12 (0.1 to 0.15)***
**r7wchange**	Weight in wave 2002 minus weight in wave 2004 (%)			0.01 (0.08)	0.04 (0.02 to 0.07)***
**r7weight**	Self-reported weight in kilograms			73.91 (16.73)	0 (-0.02 to 0.03)
**Bias variables**					
**Bias variables induced by the Functional Domains Model**					
**r7frail1_1res**	Bias induced by Dizziness as persistent problem, >=2 falls in previous 2 years, or difficulty lifting 10 pounds			0.01 (0.3)	0.19 (0.17 to 0.21)***
**r7frail1_2res**	Bias induced by Weight in wave 2002 minus weight in wave 2004 !10% of weight in wave 2002 or body mass index o18.5 kg/m2			0 (0.26)	0.15 (0.13 to 0.17)***
**r7frail1_3res**	Bias induced by Mild to severe cognitive impairment on performance-based measure or according to proxy and interviewer rating			0 (0.17)	-0.01 (-0.03 to 0.01)
**r7frail1_4res**	Bias induced by Fair or poor eyesight despite use of corrective lenses or fair or poor hearing despite use of hearing aides			0 (0.21)	0.01 (-0.01 to 0.03)
**Bias variables induced by the Burden Model**					
**r7frail2res**	Bias induced by Summary of proxy memory rating and total cognition summary score			0 (0.04)	-0.06 (-0.08 to -0.04)***
**Bias variables induced by the Biologic Syndrome Model**					

**r7frail3_2res**	Bias induced by Yes to either of two CES-D items: (i) Felt that everything I did was an effort in last week. (ii) Could not get going in last week.			0 (0.17)	-0.04 (-0.09 to 0.01)
**r7frail3_3res**	Bias induced by Frequency of three intensities of activity, lowest quintile (stratified according to sex)			0 (0.32)	0.12 (0.07 to 0.17)***
**r7frail3_4res**	Bias induced by Time to walk 8 ft, converted to time to walk 15 ft. Cutoff criteria according to sex and height remain the same			0.03 (0.49)	0.09 (0.03 to 0.14)**
**r7frail3_5res**	Bias induced by Grip strength: Weakest 20% (stratified according to sex and BMI)			0.01 (0.35)	0.03 (-0.03 to 0.08)

n = 7,713; frailty n (%) = 6,755 (87.58%); mean age = 78.43 years; female % = 58.78%.

BMI = body mass index; CES-D = Center for Epidemiological Studies Depression; HRS = Health and Retirement Study.

* = p < 0.05; ** = p < 0.01; *** = p < 0.001.

 Table 3. Frailty status defined by the Biologic Syndrome Model and its associations with frailty symptoms

HRS variables	Definitions	N with symptoms for binomial variables	Odds ratios (95% CIs	Mean (SD)	Correlation coefficients (95% CIs)
**r7agey_b**	Age at interview (years)			76.05 (7.36)	0.23 (0.19 to 0.28)***
**r7bmi**	Self-reported body mass index=kg/m2			26.41 (4.91)	-0.04 (-0.09 to 0.01)
**r7cogtot**	Total cognition summary score			21.44 (4.79)	-0.22 (-0.26 to -0.17)***
**r7effort**	Everything an effort	258	5.56 (4.04 to 7.66)***	0.16 (0.36)	0.28 (0.23 to 0.32)***
**r7frailim3**	Frailty index: Biologic Syndrome Model			1.14 (1.08)	0.73 (0.7 to 0.75)***
**r7frailim3cat**	Frailty status: Biologic Syndrome Model (outcome of this table)	203	Not applicable	0.12 (0.33)	1 (1 to 1)***
**r7going**	Could not get going	306	6.43 (4.7 to 8.8)***	0.19 (0.39)	0.31 (0.27 to 0.36)***
**r7gripl**	Grip strength, left hand			24.85 (13.1)	-0.28 (-0.32 to -0.23)***
**r7gripr**	Grip strength, right hand			27.38 (13.46)	-0.32 (-0.36 to -0.28)***
**r7height**	Self-reported height in meters			1.69 (0.1)	-0.12 (-0.17 to -0.08)***
**r7mdactx**	Frequency of moderate physical activity			2.93 (1.3)	0.41 (0.37 to 0.45)***
**r7memopr**	Proxy memory rating (1 to 6)			1 (0)	Not applicable for uniform values
**r7stroks**	had stroke since last interview (1 = no, 2 = yes)			1 (0)	Not applicable for uniform values
**r7vgactx**	Frequency of vigorous physical activity			4.18 (1.26)	0.21 (0.16 to 0.26)***
**r7walkt**	Slowness: Time to walk 8 ft, converted to time to walk 15 ft. Cutoff criteria according to sex and height remain the same			4.14 (13.29)	0.42 (0.38 to 0.46)***
**ragender**	Male = 0; female = 1	898	2.61 (1.88 to 3.64)***	0.55 (0.5)	0.14 (0.1 to 0.19)***
**Domains and frailty symptoms identified by the other 2 models**					
**r7actsum**	Summary scores of physical activities			32.41 (8.61)	0.37 (0.33 to 0.41)***
**r7arthrcat**	Binomial: Arthritis	1140	2.4 (1.63 to 3.52)***	0.69 (0.46)	0.11 (0.06 to 0.16)***
**r7bathcat**	Binomial: Problems with bathing	88	6.75 (4.3 to 10.59)***	0.05 (0.23)	0.23 (0.18 to 0.28)***
**r7cancrcat**	Binomial: Malignant disease	300	1.28 (0.89 to 1.83)	0.18 (0.39)	0.03 (-0.02 to 0.08)
**r7cogimpair**	Impaired cognition based on performance-based scores or proxy assessment	45	5.09 (2.75 to 9.42)***	0.03 (0.16)	0.14 (0.09 to 0.19)***
**r7deprescat**	Binomial: Feeling sad, blue, depressed	0	Not applicable	0	Not applicable for uniform values
**r7diabscat**	Binomial: History of diabetes mellitus	42	1.7 (0.77 to 3.72)	0.03 (0.16)	0.03 (-0.02 to 0.08)
**r7dizz**	Physical functioning: Dizziness as persistent problem	176	2 (1.34 to 2.98)**	0.11 (0.31)	0.09 (0.04 to 0.13)***
**r7dresscat**	Binomial: problem getting dressed	120	3.64 (2.39 to 5.54)***	0.07 (0.26)	0.16 (0.11 to 0.2)***
**r7eye**	Sensory problems: Fair or poor eyesight despite use of corrective lenses			2.85 (0.97)	0.13 (0.09 to 0.18)***
**r7fall**	fallen down last 2 years			0.75 (2.34)	0.11 (0.06 to 0.16)***
**r7fall_cat1**	More than 1 falls	513	2.2 (1.63 to 2.96)***	0.31 (0.46)	0.13 (0.08 to 0.18)***
**r7fall_cat2**	More than 2 falls	242	2.34 (1.65 to 3.31)***	0.15 (0.35)	0.12 (0.07 to 0.17)***
**r7frail1_1**	Dizziness as persistent problem, >=2 falls in previous 2 years, or difficulty lifting 10 pounds	573	3.3 (2.44 to 4.46)***	0.35 (0.48)	0.2 (0.15 to 0.24)***
**r7frail1_2**	Weight in wave 2002 minus weight in wave 2004 !10% of weight in wave 2002 or body mass index o18.5 kg/m2	94	8.49 (5.49 to 13.14)***	0.06 (0.23)	0.27 (0.23 to 0.32)***
**r7frail1_3**	Mild to severe cognitive impairment on performance-based measure or according to proxy and interviewer rating	45	5.09 (2.75 to 9.42)***	0.03 (0.16)	0.14 (0.09 to 0.19)***
**r7frail1_4**	Fair or poor eyesight despite use of corrective lenses or fair or poor hearing despite use of hearing aides	645	2.24 (1.66 to 3.02)***	0.39 (0.49)	0.13 (0.09 to 0.18)***
**r7frail3_2**	Yes to either of two CES-D items: (i) Felt that everything I did was an effort in last week. (ii) Could not get going in last week.	441	9.93 (7.13 to 13.84)***	0.27 (0.44)	0.38 (0.34 to 0.42)***
**r7frail3_3**	Frequency of three intensities of activity, lowest quintile (stratified according to sex)	329	17.58 (12.47 to 24.78)***	0.2 (0.4)	0.49 (0.45 to 0.52)***

**r7frail3_4**	Time to walk 8 ft, converted to time to walk 15 ft. Cutoff criteria according to sex and height remain the same	724	27.06 (14.95 to 48.96)***	0.44 (0.5)	0.38 (0.34 to 0.42)***
**r7frail3_5**	Grip strength: Weakest 20% (stratified according to sex and BMI)	290	12.72 (9.18 to 17.64)***	0.18 (0.38)	0.44 (0.4 to 0.48)***
**r7frailim1**	Frailty index: Functional Domains Model			0.83 (0.8)	0.31 (0.26 to 0.35)***
**r7frailim1cat**	Frailty status: Functional Domains Model	340	5.91 (4.34 to 8.06)***	0.21 (0.41)	0.3 (0.26 to 0.34)***
**r7frailim2**	Frailty index: Burden Model			4.17 (2.07)	0.27 (0.22 to 0.32)***
**r7frailim2cat**	Frailty status: Burden Model			0.33 (0.47)	0.19 (0.14 to 0.24)***
**r7frailim3**	Frailty index: Biologic Syndrome Model			1.14 (1.08)	0.73 (0.7 to 0.75)***
**r7grip**	Grip strength, largest value			28.31 (13.69)	-0.32 (-0.36 to -0.27)***
**r7headac**	Headache	74	1.11 (0.56 to 2.2)	0.05 (0.21)	0.01 (-0.04 to 0.06)
**r7hear**	Sensory problems: fair or poor hearing despite use of hearing aides			2.88 (1.09)	0.11 (0.06 to 0.16)***
**r7heartcat**	Binomial: Cardiac problems	562	1.83 (1.36 to 2.46)***	0.34 (0.47)	0.1 (0.05 to 0.15)***
**r7hibp**	had high blood pressure since last interview	987	1.51 (1.11 to 2.07)**	0.6 (0.49)	0.06 (0.02 to 0.11)**
**r7lift**	some difficulty in lift/carry 10lbs	440	6.83 (4.98 to 9.35)***	0.27 (0.44)	0.32 (0.28 to 0.37)***
**r7ltactx**	Frequency of light physical activity			2.71 (1.06)	0.25 (0.2 to 0.29)***
**r7lungcat**	Binomial: Lung problems	165	1.84 (1.21 to 2.79)**	0.1 (0.3)	0.07 (0.02 to 0.12)**
**r7memrysca t**	Binomial: Memory changes	20	1.79 (0.59 to 5.4)	0.01 (0.11)	0.03 (-0.02 to 0.07)
**r7mobila**	Some difficulty in mobility /05			1.12 (1.38)	0.37 (0.33 to 0.41)***
**r7muscle**	Musculoskeletal problems	57	0.53 (0.19 to 1.47)	0.03 (0.18)	-0.03 (-0.08 to 0.02)
**r7psychcat**	Binomial: Depression	165	2.38 (1.6 to 3.54)***	0.1 (0.3)	0.11 (0.06 to 0.16)***
**r7psychscat**	Binomial: Changes in general mental functioning	17	1.53 (0.43 to 5.36)	0.01 (0.1)	0.02 (-0.03 to 0.06)
**r7seizure**	Seizures, generalized	0	Not applicable		Not applicable for uniform values
**r7sleeprcat**	Binomial: Sleep changes	346	1.74 (1.25 to 2.41)**	0.21 (0.41)	0.08 (0.03 to 0.13)***
**r7strokcat**	Binomial: Cerebrovascular problems	110	2.65 (1.68 to 4.18)***	0.07 (0.25)	0.11 (0.06 to 0.15)***
**r7strokecat**	Binomial: History of stroke	139	2.56 (1.68 to 3.88)***	0.08 (0.28)	0.11 (0.06 to 0.16)***
**r7tired**	Tiredness all the time	0	Not applicable		Not applicable for uniform values
**r7toiltcat**	Binomial: Toileting problems	60	6.07 (3.56 to 10.35)***	0.04 (0.19)	0.18 (0.14 to 0.23)***
**r7underw**	Underweight in wave 2004 (%)	36	9.72 (4.95 to 19.09)***	0.02 (0.15)	0.2 (0.15 to 0.24)***
**r7urine**	Urinary incontinence	360	2.14 (1.56 to 2.94)***	0.22 (0.41)	0.12 (0.07 to 0.17)***
**r7walkt15**	Time to walk 15 feet			7.76 (24.91)	0.42 (0.38 to 0.46)***
**r7wchange**	Weight in wave 2002 minus weight in wave 2004 (%)			0 (0.06)	0.05 (0 to 0.1)*
**r7weight**	Self-reported weight in kilograms			75.75 (16.87)	-0.1 (-0.15 to -0.05)***
**Bias variables**					
**Bias variables induced by the Functional Domains Model**					
**r7frail1_1res**	Bias induced by Dizziness as persistent problem, >=2 falls in previous 2 years, or difficulty lifting 10 pounds			0 (0.29)	0.03 (-0.02 to 0.08)
**r7frail1_2res**	Bias induced by Weight in wave 2002 minus weight in wave 2004 !10% of weight in wave 2002 or body mass index o18.5 kg/m2			-0.03 (0.22)	0.21 (0.17 to 0.26)***
**r7frail1_3res**	Bias induced by Mild to severe cognitive impairment on performance-based measure or according to proxy and interviewer rating			0 (0.16)	-0.02 (-0.06 to 0.03)
**r7frail1_4res**	Bias induced by Fair or poor eyesight despite use of corrective lenses or fair or poor hearing despite use of hearing aides			0 (0.21)	0 (-0.05 to 0.05)
**Bias variables induced by the Burden Model**					
**r7frail2res**	Bias induced by Summary of proxy memory rating and total cognition summary score			0 (0.01)	-0.21 (-0.27 to -0.16)***
**Bias variables induced by the Biologic Syndrome Model**					

**r7frail3_2res**	Bias induced by Yes to either of two CES-D items: (i) Felt that everything I did was an effort in last week. (ii) Could not get going in last week.			0 (0.17)	0.08 (0.03 to 0.13)***
**r7frail3_3res**	Bias induced by Frequency of three intensities of activity, lowest quintile (stratified according to sex)			0 (0.31)	0.34 (0.3 to 0.38)***
**r7frail3_4res**	Bias induced by Time to walk 8 ft, converted to time to walk 15 ft. Cutoff criteria according to sex and height remain the same			0 (0.49)	0.3 (0.26 to 0.34)***
**r7frail3_5res**	Bias induced by Grip strength: Weakest 20% (stratified according to sex and BMI)			0 (0.34)	0.26 (0.22 to 0.31)***

n = 1,642; frailty n (%) = 540 (32.89%); mean age = 77.05 years; female % = 54.69%. HRS = Health and Retirement Study.

BMI = body mass index; CES-D = Center for Epidemiological Studies Depression; HRS = Health and Retirement Study.

* = p < 0.05; ** = p < 0.01; *** = p < 0.001.

1. Hill AB. The environment and disease: association or causation? : Sage Publications; 1965.
2. Chao Y-S, Wu H-C, Wu C-J, Chen W-C. Index or illusion: The case of frailty indices in the Health and Retirement Study. *PLOS ONE.* 2018;13(7):e0197859.
3. Chao Y-S, Wu C-J, Wu H-C, et al. Composite diagnostic criteria are problematic for linking potentially distinct populations: the case of frailty. *Scientific Reports.* 2020;10(1):2601.
4. Chao Y-S, Wu C-J, Wu H-C, et al. Using syndrome mining with the Health and Retirement Study to identify the deadliest and least deadly frailty syndromes. *Scientific reports.* 2020;10(1):1-15.
5. Cigolle CT, Ofstedal MB, Tian Z, Blaum CS. Comparing models of frailty: the Health and Retirement Study. *J Am Geriatr Soc.* 2009;57(5):830-839.
